# Relativity Theory Refounded

**DOI:** 10.1007/s10699-017-9538-7

**Published:** 2017-08-12

**Authors:** Diederik Aerts

**Affiliations:** Center Leo Apostel for Interdisciplinary Studies, Brussels Free University, Krijgskundestraat 33, 1160 Brussels, Belgium

**Keywords:** Special relativity, Refoundation, Quantum theory, Realistic interpretation, Flow of time

## Abstract

We put forward a new view of relativity theory that makes the existence of a flow of time compatible with the four-dimensional block universe. To this end, we apply the creation-discovery view elaborated for quantum mechanics to relativity theory and in such a way that time and space become creations instead of discoveries and an underlying non temporal and non spatial reality comes into existence. We study the nature of this underlying non temporal and non spatial reality and reinterpret many aspects of the theory within this new view. We show that data of relativistic measurements are sufficient to derive the three-dimensionality of physical space. The nature of light and massive entities is reconsidered, and an analogy with human cognition is worked out.

**Prologue**

She received me with warmth and led me to the dance floor in the center of the ballroom. I told her how happy I was to see her. She smiled and showed me a place at one of the tables. She asked me what I would have, adding that there were many tasty dishes. And then, in a more serious voice, she told me there was a man waiting to see me.

“The gentleman seems intent on meeting you,” she said, with a frown on her forehead, “he asked me several times whether you had arrived yet.” Then she smiled again, “I will go and tell him that you are here, and also fetch you something to eat,” and she hurried away. I saw her disappear into the crowd on the other side of the ballroom. I was sitting at the table, watching the dancing couples. Not much later she came back with all sorts of goodies for me. By her side I saw Albert Einstein, clearly recognizable by his tousled grey hair and wrinkled face.

“This is the gentleman who wants to meet you,” she said as she led him to the place at the table right in front of me. Einstein greeted me kindly and while I was still in great amazement, we began a conversation that was to last until late in the night.

*Dedicated to the cherished and sweet memory of Natalie*

## Introduction

Unlike most approaches, we will not attempt to build relativity theory (Einstein 1905a, b; Minkowski 1915; Einstein 1916, 1920, 1952; Misner et al. 1973) from as small a set of axioms as possible. Although such an axiomatic construction is very valuable, we believe that it keeps at least some of the essential aspects of ‘understanding’ hidden, because of the excessive focus on the physical content of the specific axioms that constitute a minimal set. By contrast, our presentation and analysis of relativity theory will focus from the start on those properties of the physical entities under consideration that are ‘intrinsically real’ or, using the standard terminology of relativity theory, that are ‘proper’. Further, we will make use of the mathematical structure of the theory to model specific situations and rely on the fact that this structure has been tested experimentally in innumerable ways that have proved to provide faithful models of such situations. We will also be quite frank in some of the aspects of our view and not let ourselves be misguided by new taboos arising mainly due to the intellectual struggle associated with the historical development of relativity theory—we allow ourselves to speak about ‘the flow of time’, for example. Of course, we will have to show that such a notion as ‘the flow of time’ makes sense, i.e. that we can understand its meaning within our view on relativity theory. We will also make explicit use of knowledge about the nature of reality derived from quantum theory in general and also from our conceptual interpretation of it (Aerts 2009, 2010a, b, 2013, 2014). As we shall see, it is in a conversation with relativity theory as it is usually perceived, taking into account insights from quantum theory and also being open to intuitions beyond, which allows us to put forward a new view of the physical reality described by relativity theory, giving rise to a possible understanding of the deep conceptual problems of interpreting the nature of this physical reality.

## Intrinsic Aspect of Physical Entities

An important difference with the traditional view on relativity is that we will consider physical entities not a priori as ‘material objects occupying a specific time–space region inside a universe that is also inside time–space’. We rather want to consider time–space as a ‘theatre of encounter for physical entities’. This is the reason why we make an attempt to build the theory starting as much as possible from what we have learned from traditional relativity theory—its classical framework as well as the known quantum versions—to be the intrinsic aspects of such physical entities.

As we will see, in our view, for a specific physical entity the flow of time exists, and contrary to what is often believed, this flow of time is intrinsic. It is what is called in relativity theory ‘the proper time of a physical entity’. Using the notation for proper time in relativity theory, we denote this time by the Greek letter $\tau$. This time can be measured by any sufficient regular repetitive part of the physical entity we consider, let’s call such a repetitive part a clock. As we will develop our new foundation of relativity theory step by step in the present article, we will become much more specific about the physical meaning of the statement ‘the flow of time exists’, and see that it actually means that ‘the flow of the landscape of a non temporal reality exists and for each entity its time parametrises this flow’. A physical entity, if not a point particle, has an extension, i.e. takes a place while it also takes a time. Also this extension can be given an intrinsic measure, depending on the specific geometric structure of the physical entity, it can be specified by giving the sizes of the lengths separating different points needed to characterize this geometric structure. We measure these lengths by what in relativity theory is called the ‘proper length’. We will denote this length, taking over the common notation for relativity theory, by the letter *s*. In fact, as long as we consider one unique specific physical entity, not much more can be said, it has an intrinsic extension—including a specific geometric form—and an intrinsic elapsing of its time.

Of course, reality consists of more than one physical entity, hence let us consider two such entities *A* and *B*, both equipped with clocks that measure their respective proper times $\tau ^A$ and $\tau ^B$, and also equipped with two extensions measured by the proper lengths $s^A$ and $s^B$. We want to start considering the simplest of all imaginable situations, but one for which we know that it exists in our reality, which is the following. Both physical entities *A* and *B* are at rest with respect to each other. We have not yet specified anything about the nature of the physical entities, and will do so now, just to make the situation easier to imagine. So, we suppose that both entities are pieces of matter with masses $m^A$ and $m^B$ and we indicate by $(x_{1}^A, x_{2}^A, x_{3}^A)$ and $(x_{1}^B, x_{2}^B, x_{3}^B)$ their centers of mass, respectively, indicating their places—by introducing three numbers to indicate these centers of mass, we already prelude the fact that we will start considering more complex situations where at least one of the physical entities moves with respect to the other, and kind of also prelude that there are three orthogonal dimensions describing how such a movement can take place, but these are not essential elements, and rather introduced here to keep our analysis simple enough to be able to focus on the core of the matter.

Hence, as we said, *A* and *B* have places indicated by points $(x_{1}^A, x_{2}^A, x_{3}^A)$ and $(x_{1}^B, x_{2}^B, x_{3}^B)$ and these points do not move with respect to each other—and this is also the case for any other two points we would have chosen to identify the places of *A* and *B*. Another way of stating the same would be to say that *A* is at rest with respect to *B* and *B* is at rest with respect to *A*. We know from experience that this is possible—we will later see that when the effects described by general relativity become important, we have to be more careful in defining well this type of situation, but for the time being, this is how we start. In such a situation, *A* and *B* can easily synchronize their clocks, and from relativity theory follows that if they do so, both clocks measure the same proper or intrinsic time. This means that we can put in this situation $\tau ^A=\tau ^B$. Also for the measuring of length, *A* and *B* can use the same measuring rod, and agree about all necessary aspects of the procedure of length measuring, and again, relativity theory teaches us that $s^A=s^B$ in this case of *A* and *B* being at rest with respect to each other. This means that *A* and *B* can measure an absolute distance that separates them. Let us make very clear, however, at this stage, because here the customary confusion in most interpretations of relativity theory already starts, ‘what we have identified as being intrinsic’ and also ‘what we have not identified at all’. We have identified that both *A* and *B* have the same intrinsic flow of time, hence $\tau ^A=\tau ^B$, and they can synchronize their clocks, which will remain synchronized as long as *A* and *B* remain in this way at rest with respect to each other. We have also identified that *A* and *B* can measure with the same intrinsic length, hence $s^A=s^B$, and in this way can determine an intrinsic length that separates them. What we have not done is to identify a time–space where both are present, such that their time would be the time elapsing in this time–space and their lengths would be measured by the distances of this time–space. This, i.e. the identifying of such a time–space, we have ‘not’ done. And, as we will see in the analysis we will make now, it is something we should only do with extreme care.

The next situation we want to analyze, taking into account our knowledge of relativity theory, theoretical as well as experimental, is where one of the two physical entities starts to move with respect to the other. Let us say that *B* starts to move with respect to *A*, and *A* remains without moving—we will come later to specifying more carefully still what we mean by this. To make the calculation simpler, we suppose that *B* moves with constant velocity equal to $0.9\, c$, where *c* is the velocity of light, in the direction $x_1$. Let us make still more explicit what we mean exactly by this. We mean that the intrinsic distance between *A* and *B*, measured by *A*—hence the intrinsic distance for *A*, and not for *B*—increases in size with the time flow of *A*—hence the flow of the intrinsic time of *A* and not of *B*—in a constant way, for example in one second the distance has increased of 0.9 light second, a light second being the distance traveled by light in 1 second. Relativity teaches us that the ‘time flow’ of the moving *B*—hence the intrinsic time of *B* and not of *A*—will slow down substantially, and we can exactly calculate how it slows down. Let us become concrete, and suppose that *A* remains without moving from time $\tau ^A=0$, to time $\tau ^A=10$ years. In these 10 years, moving with a velocity equal to $0.9\, c$, *B* has moved away from *A* over a distance $\Delta x=9$ light years. Then the time $\tau ^B$ will have elapsed an amount equal to

1 $$\begin{aligned} \Delta \tau ^B=\sqrt{\left( {\Delta \tau ^A}\right) ^2-\left( {v\Delta \tau ^A \over c}\right) ^2}\approx 4.3589\ \mathrm{years} \end{aligned}$$

So, less than half of the time has elapsed for *B* as compared to the time that has elapsed for *A*.

Immediately two questions arise. The first question, the one of the famous twin-paradox, is the following. Why would we not look at the situation the other way around, and consider *B* to be not moving while *A* is moving in the opposite direction? If we were allowed to make this ‘relativistic’ symmetry reasoning, we could as well come to the conclusion that time has elapsed more slowly for *A* instead of for *B*. A first—but, as we will see, incorrect—answer would be the one that comes to mind right away, namely the following. Only one of the two ‘starts’ to move, and hence undergoes during a certain time an acceleration for speeding up to the constant velocity, and the other, since remaining at rest, does not experience this acceleration. The presence of this acceleration breaks the symmetry of the situation. We can, however, making the situation slightly more complex, overcome the necessity of having an acceleration involved. Indeed, suppose that *B* was moving all the time with constant velocity, and both *A* and *B* just took their encounter as an opportunity to synchronize their clocks, in principle this is possible, and then the situation is indeed completely symmetric. For such a situation, where *B* was already moving, we indeed can equally well decide that *A*’s clock is slowing down, or even that both clocks are behaving still in a more complicated way with respect to each other. We will come back to this in detail after a further analysis, which we will make right away.

Indeed, we can only decide about the problem just mentioned if we configure further the situation in such a way that *A* and *B* will meet again in their common future. Suppose this happens by ‘only’ *B* having to stop moving away from *A*, turning around, and start moving towards *A* with the same velocity. Then, after 20 years have passed for *A*, they will meet again, and only 8.7178 years will have passed for *B*. Does the asymmetry comes now from the fact that *B* needs to experience an acceleration during the turning around? Again, this is not the case. We could involve a third physical entity *C*, which encounters *B* exactly after 4.3589 years have passed for *B*, synchronizes clocks, and then moves towards *A* with the inverse velocity of *B*. Then *C* will encounter *A* after 4.3589 years exactly, carrying a message from *B* who left *A* 8.7178 years ago on *C*’s clock. And on *A*’s clock 20 years will have passed. So, it is not the effect of the presence of an acceleration which causes the ‘slowing down of time flow’. It is the geometric structure of the paths crossed by *A*, *B* and *C* which contains the fundamental asymmetry that is predicted by relativity theory and also has been observed on numerous occasions experimentally.

By the way, the above analysis also makes clear that it is not correct to interpret the difference between the passage of time for *A* as compared to *B*—suppose for a moment we forget again about *C*, and hence are considering the situation where *B* turns around and after the turn heads back towards *A*—as due to a physical effect that, for example, would be forced upon the mechanics of the clock of *B* as a consequence of the acceleration during the turning around. There is no ‘physical mechanical effect on clocks’ involved in the time dilatation effect of relativity theory. The difference between $\tau ^A$ and $\tau ^B$, and let us repeat that ‘both’ are intrinsic times for the physical entities *A* and *B*, respectively, is due to *A* and *B* having travelled a different path in the time–space structure of relativity theory. Moving, what *B* does with respect to *A*, does not only give rise to ‘moving in space’, but also simultaneously to ‘moving in time’. Well, if only for a moment, in what we just wrote, we have given into the desire of considering a global time–space structure in which *A* and *B* would be present and could move around. Let us give into this desire deliberately now, such that we can see which are the deep paradoxes that arise from it.

If there is a time–space continuum, for special relativity—which contains the core of the mystery—this would be the Minkowski time–space, in which four vectors are intrinsic entities. Then, as we concluded already above, the difference in elapsed time between a moving physical entity such as *B* and an entity at rest such as *A*, is due to *B* and *A* taking a different path through time–space to travel from the event where they first encounter to the event where they encounter for the second time. The path of *A* is a path that connects the two time–space events $(0, x_1^A, x_2^A, x_3^A)$ and $(20\ \mathrm{years}, x_1^A, x_2^A, x_3^A)$ by means of a straight line parametrized as $(x_0^A, x_1^A, x_2^A, x_3^A)$, where $x_0^A$ runs from 0 to 20 years. The intrinsic time $\tau ^A$ which passed for *A* is calculated from the general formula of Minkowski time–space given by

2 $$\begin{aligned} \Delta \tau =\sqrt{(\Delta t)^2 - \left( {\Delta x_1 \over c}\right) ^2 - \left( {\Delta x_2 \over c}\right) ^2 - \left( {\Delta x_3 \over c}\right) ^2} \end{aligned}$$

Given that for *A* we have $\Delta x_1=\Delta x_2=\Delta x_3=0$ light years and $\Delta t= 20$ years, we get

3 $$\begin{aligned} \Delta \tau ^A=\sqrt{(\mathrm{20\ years})^2}=\mathrm{20\ years} \end{aligned}$$

The path of *B* is a very different one. It first connects, also in a straight line, the points $(0, x_1^B, x_2^B, x_3^B)$ and $(10\ \mathrm{years}, x_1^B+9\ \mathrm{light\ years}, x_2^B, x_3^B)$, with $x_1^B=x_1^A$, $x_2^B=x_2^A$ and $x_3^B=x_3^A$. Then, after *B* has turned around to head again towards *A*, it connects, again in a straight line, the points $(10\ \mathrm{years}, x_1^B+9\ \mathrm{light\ years}, x_2^B, x_3^B)$ and $(20\ \mathrm{years}, x_1^B, x_2^B, x_3^B)$. If we calculate by means of the same intrinsic definition for time in Minkowski space the time elapsed for *B* on these two paths, we find, for both paths $\Delta x_1=9$ light years, $\Delta x_2 = \Delta x_3 = 0,$ and $\Delta t = 10$ years. Hence the time elapsed on each path is given by

4 $$\begin{aligned} \Delta \tau ^B (\mathrm{each\ path\ for\ } B)=\sqrt{(\mathrm{10\ years})^2 - \left( {\mathrm{9\ light\ years} \over c}\right) ^2}\approx 4.3589\ \mathrm{years} \end{aligned}$$

and therefore, the total time elapsed for *B* is

5 $$\begin{aligned} \Delta \tau ^B (\mathrm{total\ path\ for\ } B)\approx 8.7178\ \mathrm{years} \end{aligned}$$

So, indeed, we see that the time difference is due to the difference between the path taken by *B* as compared to *A* to encounter each other in the two events $(0, x_1^A, x_2^A, x_3^A)$ and $(20\ \mathrm{years}, x_1^A, x_2^A, x_3^A)$ for the case of *A*, and $(0, x_1^B, x_2^B, x_3^B)$ and $(20\ \mathrm{years}, x_1^B, x_2^B, x_3^B)$ for the case of *B*. And, this difference is due to the structure of Minkowski time–space as a four-dimensional manifold.

Hence, we have to conclude, at this point of our analysis, that the geometric interpretation of relativity holds, namely that ‘time–space’ is what intrinsically exists, while ‘time’ and ‘space’ as separated entities cease to exist. Customarily this is called the block universe interpretation of relativity. It comes along, however, with severe trouble. In case reality ‘is’ the four-dimensional time–space of Minkowski, what is then the meaning of ‘change in time’? Does this mean that physical entities exist within the four-dimensional time–space manifold, and are their world lines? And when a human being experiences a physical entity, does he or she experience a ‘slice’ in the time–space continuum of this physical entity’s world line? But, if this was the case, why are we as individuals not four-dimensional? We definitely are not our past and future all at once. Does this then mean that it is our consciousness that in some way ‘travels’ on the world line which our body is, the latter being four-dimensional? Would this also mean that for what concerns physical reality change is not possible, and is only an illusion provoked by our consciousness, while the future is as fixed as the past and the present?

Or, do we have to go to the other extreme, and affirm that existence is not four-dimensional at all? And is Minkowski time–space only a mathematical construction, and is it such that all physical entities just travel on their world lines? The problem with this view is that, and this follows from the reasoning we described above, the relativistic effects really come about as a consequence of the different paths connecting time–space points in the four-dimensional Minkowski manifold. They cannot be retrieved as due to effects on individual physical entities that travel on paths, because they are due to the way such a path is part of the global four-dimensional manifold. Hence, we can say that the situation is truly characterized by something related to ‘four dimensions’, and that any attempts to escape this are bound to fail.

Opinions are divided about these two options, and nobody, as far as we know, seems to really understand the situation. We believe that the reason is that both views are wrong, and it is a third view, quite different from the two encountered in the literature which is the correct one. It is this third view that we want to start to elaborate here.

## The Reality Beneath Space–Time

The view that we propose needs insight from quantum mechanics, and hence relies on the fact that relativity theory without quantum mechanics is not complete. More specifically, it relies on the creation-discovery view that we developed for quantum mechanics and the hypothesis to interpret quantum non-locality as quantum non spatiality (Aerts 1998, 1999). We go a step further, however, and put forward the hypothesis that physical entities are intrinsically non spatial and also non temporal. And that ‘time’ and ‘space’ are consequences of a creation aspect within our creation-discovery view.

Of course, like the saying goes, ‘the proof of the pudding is in the eating’, and hence we have to show that the above-mentioned hypothesis makes sense. How better to provide such proof than by giving a simple example that enables us to directly see and understand what could be the relevance of such hypothesis for our physical reality? The example is inspired by our ‘conceptuality interpretation of quantum mechanics’ (Aerts 2009, 2010a, b, 2013, 2014), although this does not mean a priori that it is also an argument in favor of this interpretation. Here, we only want to show how the view on relativity theory—and hence also on quantum mechanics with non spatiality and non temporality—that we put forward can be true, and what are some of its immediate consequences.

Our example consists in considering a definitely non spatial and also non temporal collection of entities, namely the conceptual meaning structure of humanity. In a first stage, to be as concrete as possible, we consider it in the form of the World-Wide Web. How do we find a time–space structure connected to the World-Wide Web? Well, each time that we log into the World-Wide Web using our computer and our browser, and we see in front of us a specific webpage, we consider this as ‘an instance’ of conceptual meaning of the World-Wide Web, where the meaning content of that webpage ‘takes time and place’, hence ‘becomes localized in time and space’. Indeed, also a specific instant of time, for example connected to the click of our mouse on the link given to us by Google after a search, and the opening of the webpage, is connected to it. Hence, the happening is an ‘instantiation’ of non spatial and non temporal conceptual meaning part of the World-Wide Web. If we push on a specific link that we see on the webpage, we move to another webpage, and also to another meaning content which at that time gets localized on the newly appeared webpage. We could have pushed another link, and this would have brought us to usually another webpage, possibly to the same, however. Anyhow, we all know very well the dynamical process we put forward here, it is called ‘surfing the World-Wide Web’. Suppose we consider two persons *A* and *B* surfing and starting from the same webpage with a mutual experience that they ‘meet before starting both their surfing’, and such that, after having taken a different path through the World-Wide Web, they also end up at the same webpage in a mutual experience that ‘they meet again’. So they started together and then meet again. Quite obviously, if we measure intrinsic time for both with the repetitive actions of their clicking of new links, this time in general will be quite different for *A* and *B*, and exactly depending on the paths that both have followed to get from the first starting webpage to a second webpage where they meet again. The difference in time is intrinsically due to the structure of the World-Wide Web, and the different paths that can be taken to go from one webpage, where *A* and *B* meet in the beginning, to another webpage, where *A* and *B* meet the second time.

The foregoing scenario is realistic, i.e. it can be realized, as is clear that by counting the number of clicks that both paths need to meet again at the same webpage, we can easily calculate the difference of the flow of time for *A* and *B*. The reason that it can happen is because the World-Wide Web represents a coherent collection of meaning. We can also understand now what is the meaning of ‘flow of time’, it is actually the ‘passing from one instance to another instance’, where instances are ‘instantiations’ of an underlying non spatial and non temporal conceptual meaning, i.e. happenings that bring this underlying non spatial and non temporal conceptual meaning to a time–space coordinate of the one for whom ‘time flows’. We can add some additional aspects to give our example more explicative power. For example, every webpage could have some links that direct to the same webpage. If both *A* and *B* proceed with these links, they will follow two paths with times that are equal, at least if they synchronize their clicking speed—but that is also necessary in physical reality. Pushing another link, in the analogy, would mean already ‘no longer being at rest with respect to each other’. It will be completely determined by the structure of the underlying non spatial and non temporal World-Wide Web, how the different paths of *A* and *B* will fare in between them reaching the same webpage again at the same time, and hence meeting again. But, the time that elapses for both will be intrinsic, because totally determined by the structure of the underlying non spatial and non temporal World-Wide Web. Namely the time will be completely determined by the number of webpages lying in between, the number of places that are encountered to move over this specific path from the first meeting spot to the second meeting spot.

Can we understand by means of this example that we do not get into the trouble of the block universe interpretation? Indeed, it follows from the example that there is a real past, which has passed, and there is a present—although only locally for one entity—and there is a future which is not fixed. What is however given and existing outside of the time measured by the clicking of surfers is the meaning structure of the World-Wide Web. This is not a four-dimensional time–space structure, because it is non spatial and non temporal, but its realization as a consequence of surfing it, does get related in a specific way to such a four-dimensional manifold structure. It is also clear that the ‘lapsing of time’ gives rise to a dimension which is structured as if it were a space dimension, but this does not mean that this time-dimension ‘is’ a real existing piece of reality. The structure of the time-dimension follows from the structure of the non temporal and non spatial World-Wide Web, and indeed ‘how the space dimensions get structured’ is equal to ‘how the time dimension gets structured’, while what is underlying is always the structure of ‘how meaning is coherently connected’—we will analyze later how ‘space appears for the example of the World-Wide Web’.

If we apply this explanation to physical reality, does this still lead to physical entities being four-dimensional world lines? No, but it does mean that physical entities extend their coherent nature into their future and their past too, like they do in the space directions—and a space direction means for a physical entity ‘those places where another physical entity might be’. But ‘it is not an already determined future’ while ‘it is a past that has already passed’. Hence, it is not that ‘they exist in the future’, but only that observers use links that were already inside the meaning structure of physical reality when making their path to the future. But the parts of the already existing reality, which is non temporal and non spatial, like the meaning content of the World-Wide Web is, have no ‘time dimension’ connected to it, ‘before the surfing action has chosen which path to take. If we define ‘happenings’ as ‘meaning parts of the World-Wide Web—hence no surfing yet involved—then the ‘taking time and place’ is the ‘creation’ aspect of a creation-discovery dynamics. Time–space is not discovered but created, but a vast underlying non temporal and non spatial structure ‘is’ present, in our example it is the meaning structure of the World-Wide Web. Let us also mention that the World-Wide Web in itself also changes constantly, but this change is not related to the change taking place while surfing. Hence, this could be the same for physical reality, which changes constantly, but this change is not essentially related to the change we see if we surf it wearing space–time clothes.

It is, by the way, here that we would like to add an aspect that is not present in the World-Wide Web, but that would have been present if we had considered a deeper version of the meaning structure of the human realm. In the paths followed by physical entities also direct creation of meaning is possible. In the example of the World-Wide Web only already existing meaning and existing links can be followed—albeit also the World-Wide Web is evolving constantly towards a more rich structure and quite some aspects of it incorporate already the possibility of direct creation of meaning. Hence, in this respect the example should be enriched to capture the more quantum nature of physical reality, and then the two types of change mentioned above would be linked. But that was not the aim of it here, the aim rather being to show that with this example we can understand relativity theory.

Let us analyze what the equivalent of ‘space’ is within this view. The ‘space’ surrounding a physical entity is the collection of ‘places’ where other physical entities can be together with the considered physical entity, and we use here the term ‘be’ in a non temporal and non spatial sense, where ‘temporal’ relates to the ‘time elapsing for each individual physical entity’ while surfing.

Without being very explicit about it, we already used aspects of the notion of space when we reasoned about *A* and *B* being ‘at rest’ with respect to each other. We have to try to see clear in these notions now for our example of the World-Wide Web. Suppose we consider a webpage $A_0$ that we open on our computer, then obviously there is an enormous amount of other webpages that ‘we could have opened’ but we did not. They all are webpages that we could have opened if we had made another decision in the past of the procedure of actually opening webpage $A_0$ (Aerts 1996a, b). Also, someone else could actually be looking at the same webpage simultaneously and then surf in another direction. To introduce more specific aspects of these ‘space-like’ situations and do this in such a way that the comparison with the situation of physical entities in Minkowski space can be meaningfully made, we proceed as follows.

We suppose that ‘surfing’ is continuously happening. So, when we consider a snapshot of it, for example, webpage $A_0$ that is looked at, we suppose that the webpage was realized by clicking a link of another website, that was open ‘before’ $A_0$ was open. And then it continues, after $A_0$ being open, another webpage will be open by clicking a link on $A_0$. Let us also mention here that in our example, surfing happens actively due to a human being, however it is not Einstein’s ‘observer’ the role played by the human being, in case we would go from our example to the situation of the physical world. It is the physical entities themselves that play the equivalent of the role of surfing, and that hence ‘create’ parts of time–space when being what they are. That is why in the opening text of this investigation we mentioned that we will put forward time–space as a theater of encounter of physical entities.

Next to single webpages, we will introduce world lines which are ‘sequences’ of webpages $(A_n)_n$, where *n* runs from 0 to the natural number *m*. So, more concretely, the world line $(A_n)_n$ consists of the set $\{A_{0}, \ldots , A_{m}\}$ of webpages surfed through one after the other, and the world line $(B_k)_k$ consists of the set $\{B_{0}, \ldots , B_{l}\}$ of webpages, surfed through one after the other.

Let us consider $A_0$. For the concrete world line $\{A_0, \ldots , A_m\}$, the webpage $A_0$ contains numerous other links that could have been pushed instead of the link that leads to the realization of the world line $\{A_0, \ldots , A_m\}$. Let us suppose now that $(A_n)_n$ and $(B_k)_k$ start of at the same webpage $A_0=B_0$ but with other links being pushed which means that they go off in different directions after starting from the same webpage. A possible way is that a second person *B* surfs through the world line $\{B_0, \ldots , B_l\}$, and starts his or her surfing together with person *A* at the same webpage. Additionally, we suppose that both also end at the same webpage, hence $A_0=B_0$ and $A_m=B_l$. In general *m* will be different from *l*, and if we consider *m* and *l* respectively as measures of the intrinsic times that passed by when *A* and *B* ran through their respective world lines, this would mean that different intrinsic times passed by for *A* and *B*. We can consider the set of all world lines that start at the same webpage $A_0$ and end at the same webpage $A_m$, and call this set ${{\mathcal {A}}}_{0,m}$.

## Minkowski Coordination

We are now ready to introduce a Minkowski type coordination for ${{\mathcal {A}}}_{0,m}$. We proceed as follows. We choose a time-axis that coordinates the elements $A_n$ of the world line $(A_n)_n$ at equal spaced points on the axis, and without loss of generality, since it consist only in fixing a unit time interval, we choose the coordinate numbers $0, \ldots , m$ for the elements of $(A_n)_n$, where for simplicity we don’t write explicitly the time units. Let us consider now the world line $\{B_0, \ldots , B_l\}$ element of ${{\mathcal {A}}}_{0,m}$. We know that $B_0=A_0$ and $B_l=A_m$, which means that we will coordinate $B_0$ and $B_l$ by the same values 0 and *m* of the introduced time axis. What about the other webpages, starting with $B_1$ and continuing to $B_{l-1}$? It is here that ‘space’ comes into being as a way to give a place to webpages that exists together with the ones that we have coordinated already on the time axis. Of course, at once this means that we also give a place to the elements of $(A_n)_n$, namely, ‘they will be at rest in the origin of the coordinate system that we put up’. This means that an entity *B* associated with the elements of $(B_k)_k$ will not be at rest, but moving. But, all this needs to follow from our careful operational construction, so let us proceed step by step.

An arbitrary element of $(B_k)_k$, for example the element $B_k$, will be given a time–space coordinate indicating its place as an element of the set ${{\mathcal {A}}}_{0,m}$ and of the time–space coordinate system that we introduce, in the following way. Consider the time interval $[t_0(A), t_m(A)]$ where, $t_0(A)$ and $t_m(A)$ are the time coordinates of $A_0$ and $A_m$ respectively. Then all other time coordinates of elements of $(A_n)_n$ are points inside $[t_0(A), t_m(A)]$ of equal distance from each other. We choose $t_0(B)=t_0(A)$ and $t_m(A)=t_l(B)$, and divide the interval $[t_0(A), t_m(A)]$ in *l* equal parts to give time coordinates to all other points of $(B_k)_k$. However, contrary to all elements of $(A_n)_n$ having space coordinate equal to zero, this is no longer the case for the coordinates of the elements of $(B_k)_k$ different from $B_0$ and $B_l$. Let us explain how we coordinate all elements of $(B_k)_k$. To $B_0$ we give the time–space coordinate $(t_0(B), 0)=(t_0(A),0)$, expressing that both $(A_n)_n$ and $(B_k)_k$ take off as world lines at the same websites $A_0=B_0$. Without loss of generality, we can choose $t_0(A)=t_0(B)=0$, which consists of letting the origin of the time–space coordinate system—which for the time being it is sufficient to consider being two-dimensional—be at the beginning webpage for both world lines. Again without any loss of generality we can take $t_1(A)$ as our time unit in the considered reference frame and set $t_1(A)=1$.

We introduce a positive number *c*—it will play the role of the velocity of light in our example—but postpone the analysis of its meaning to later. We will make now a choice for the time–space coordinate of the webpage $B_1$ which only depends on this number *c* and on the numbers *m* and *l* which is the following

6 $$\begin{aligned} \left( t_1(B\right) , x_1(B))=\left( {m \over l}, c{\sqrt{m^2-l^2 \over l^2}}\right) \end{aligned}$$

and will explain why this is the choice to be made if we want to coordinate within the Minkowski metric the path of *B* in the following simple way. First *B* moves away from *A* with constant velocity *v* , which is a scalar quantity in this reasoning, and then halfway it turn back to *A* with the same velocity *v*. Let us calculate $t_1(B)$ and $x_1(B)$ in this situation. Since also the clicks on the path of *B* are equal, we have $t_1(B)={l \over m}t_1(A)={m \over l}$. To calculate $x_1(B)$ we note that if *c* equals the velocity of light, then it follows from the time dilatation formula that

7 $$\begin{aligned} {l \over m}=\sqrt{1-{v^2 \over c^2}} \end{aligned}$$

From this follows that

8 $$\begin{aligned} {l^2 \over m^2}={1-{v^2 \over c^2}} \Leftrightarrow 1-{l^2 \over m^2}={v^2 \over c^2} \Leftrightarrow \sqrt{1-{l^2 \over m^2}}={v \over c} \end{aligned}$$

which shows that *B* will move with constant velocity

9 $$\begin{aligned} v=c\sqrt{1-{l^2 \over m^2}} \end{aligned}$$

in the space realm of the time–space coordinate system of *A*. This means that

10 $$\begin{aligned} x_1(B)=vt_1(B)=c{m \over l}\sqrt{1-{l^2 \over m^2}}=c{\sqrt{m^2-l^2 \over l^2}} \end{aligned}$$

We want to remark here that the above calculations are only meant to explain why we choose (6) for the time–space coordinate of $B_1$, and it is important to note that only the values of *m*, *n* and the value of a positive quantity *c* is needed for this choice. If we consider additionally *B* to move with constant velocity *v* away from *A* in the $x_1$ space direction of the coordinate system of *A*, then *v* is given by (9).

Let us analyze this. The fraction of the velocity of light *c* that determines the velocity *v* is given by $\sqrt{1-{l^2 \over m^2}}$. This means the following. When a link is chosen, then $\sqrt{1-{l^2 \over m^2}}$ stands for the velocity through space, expressed as a fraction of the velocity of light, which is the maximum velocity through space which is possible, that this links carries with itself related to the purpose of ‘reaching the end webpage’, where meeting is taking place.

Let us look at the two extremes. When $l=m$, then this velocity is zero. Indeed, it means that the chosen road is equally slow as the slowest one, which is $(A_n)_n$. Since we directed our time axis along the latter, space is only involved in a passive way, i.e. no movement is space takes place. Consider the other extreme, namely $l=0$. This means that ‘no link is needed to go from the begin webpage to the end webpage’. In fact, this is a ‘limit’ situation not possible to reach by surfing, where the fastest way would be ‘one click’, hence ‘one link’. But anyhow, in this situation of zero links, we get that $v=c$, namely that we need to move through space with a velocity equal to that of light.

Let us construct now the coordinates of all the elements of $(B_k)_k$. We make the additional hypothesis that both *m* and *l* are even numbers—the uneven case needs a slightly different treatment, but is essentially completely analogous, hence we leave its details to be worked out by the interested reader. The following are then the time–space coordinates of all elements of $(B_k)_k$.

11 $$\begin{aligned} B_0 \leftrightarrow \left( 0, 0\right) \end{aligned}$$

12 $$\begin{aligned} B_1 \leftrightarrow \left( {m \over l}, c\,{\sqrt{m^2-l^2} \over l}\right) \end{aligned}$$

13 $$\begin{aligned} & B_2 \leftrightarrow 2\, \left( {m \over l}, c\,{\sqrt{m^2-l^2} \over l}\right) \\ &\qquad \vdots \end{aligned}$$

14 $$\begin{aligned} B_{{l \over 2}-1} \leftrightarrow \left( {l \over 2}-1\right) \left( {m \over l}, c\,{\sqrt{m^2-l^2} \over l}\right) \end{aligned}$$

15 $$\begin{aligned} B_{l \over 2} \leftrightarrow \left( {m \over 2}, c\,{\sqrt{m^2-l^2} \over 2}\right) \end{aligned}$$

16 $$\begin{aligned} & B_{{l \over 2}+1} \leftrightarrow \left( {m \over 2}, c\,{\sqrt{m^2-l^2} \over 2}\right) +\left( {m \over l}, -c\,{\sqrt{m^2-l^2} \over l}\right) \\&\qquad \vdots \end{aligned}$$

17 $$\begin{aligned} B_{l-1} \leftrightarrow \left( m-{m \over l}, c\,{\sqrt{m^2-l^2} \over l}\right) \end{aligned}$$

18 $$\begin{aligned} B_l \leftrightarrow \left( m, 0\right) \end{aligned}$$

**Fig. 1:**
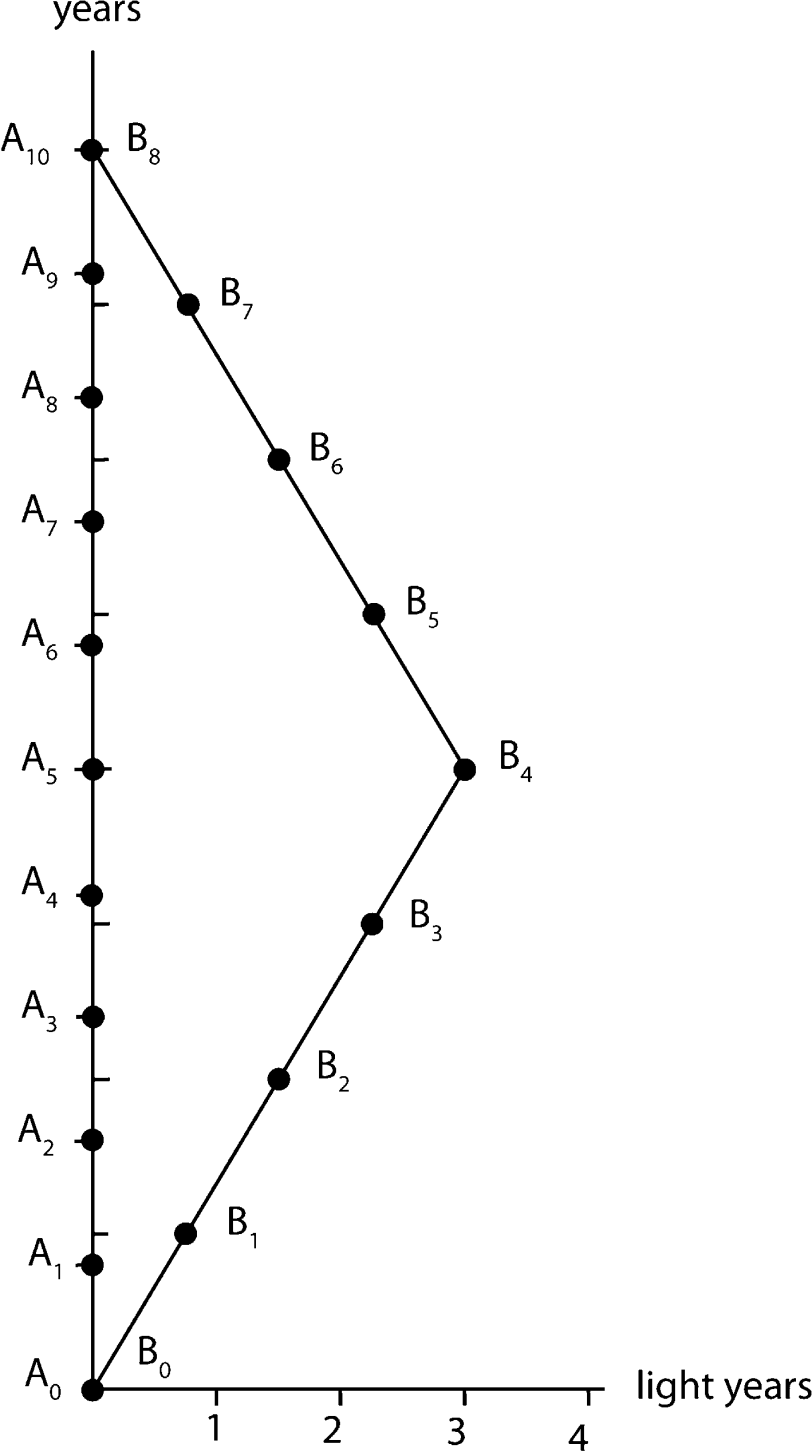
A graphical representation of *A* and *B* for $m=10$ and $l=8$

In Fig. 1 we have graphically illustrated the situation for $m=10$ and $l=8$. And with units of time in ‘years’ and unit of length in ‘light years’. Hence, in the situation represented in Fig. 1, we have

19 $$\begin{aligned} B_0 \leftrightarrow (0, 0) \end{aligned}$$

20 $$\begin{aligned} B_1 \leftrightarrow \left( {5 \over 4}, {3 \over 4}\right) = (1.25,0.75) \end{aligned}$$

21 $$\begin{aligned} B_2 \leftrightarrow \left( {5 \over 2}, {3 \over 2}\right) = (2.50, 1.50) \end{aligned}$$

22 $$\begin{aligned} B_3 \leftrightarrow \left( {15 \over 4}, {9 \over 4}\right) =(3.75,2.25) \end{aligned}$$

23 $$\begin{aligned} B_4 \leftrightarrow (5, 3) \end{aligned}$$

24 $$\begin{aligned} B_5 \leftrightarrow \left( {25 \over 4}, {9 \over 4}\right) =(6.25,2.25) \end{aligned}$$

25 $$\begin{aligned} B_6 \leftrightarrow \left( {15 \over 2}, {3 \over 2}\right) =(7.5,1.5) \end{aligned}$$

26 $$\begin{aligned} B_7 \leftrightarrow \left( {35 \over 4}, {3 \over 4}\right) =(8.75,0.75) \end{aligned}$$

27 $$\begin{aligned} B_8&\leftrightarrow&(10, 0) \end{aligned}$$

Like can be seen on Fig. 1, we have constructed the world line for *B* in such a way that *B* first moves with velocity $v=c\sqrt{1-{l^2 \over m^2}}\approx 0.6\,c$ in the positive direction of the *x*-axis, hence moves ‘away’ from *A*. Then halfway – which is the reason that we introduced the hypothesis for *m* and *l* to be even numbers—*B* turns around, and starts to move with the same magnitude of velocity $v=c\sqrt{1-{l^2 \over m^2}}\approx 0.6\,c$, but in the opposite direction, hence approaching *A* again. This is the reason that both can meet again at $A_m=B_l$. Meanwhile however $m=10$ years have passed by for *A*—or *A* has clicked $m=10$ webpages while surfing—and only $l=8$ years have passed by for *B*—or *B* has clicked $l=8$ webpages while surfing. Everybody can easily recognise the typical situation considered in the twin paradox of special relativity theory.

There are different reflections to be made. First of all, the representation that we gave for $(B_k)_k$ is not general, there are many different possible configurations that realise within a Minkowski metric the situation $l \le m$. What is however interesting to remark is the following. In case we limit ourselves to ‘constant velocities and movements in straight lines’—possibly with a brusque turn around like in our example—in principle all possibilities are equivalent.

Indeed, it is always also necessary to make at least a turn around, if we start by representing the slowest path by a straight line without any turn around. What is the meaning of this? We have to invoke general relativity perhaps to be able to understand better. Indeed, instead of straight lines, a constant velocity and a brusque turn around, it would be possible to realise the path of *B* by means of a curved line, were accelerations play a role. And, one could switch completely to general relativity. The slowest path—hence *A*—would then be the path following a geodesic of the gravitational situation. It is more easy to understand that ‘this geodesic’ path is the one we should give preference in coordinating, of course, as in the case of general relativity the coordination would only locally be Minkowskian. What needs to be mentioned is that even then the counter intuitive aspect—which we will try to formulate better and analyse deeper right away—remains present, because locally the Minkovski metric governs, which means that time–space is curved hyperbolically.

This hyperbolic curvature of Minkowski space means that ‘velocity, even if we identify it at first hand as a movement through space’ is mainly a movement through time, i.e. along the axis of time. However, like our example of the World-Wide Web and surfing shows, this does not mean that we need to go towards a block interpretation of reality. What does it mean then? Well, it means that we can influence the way we reach out in the future by getting ourselves moving, and if we accelerate the influence becomes even bigger—we literally move faster and a time coordination will reveal this. If, instead, we let ourselves float on a geodesic we move in the slowest possible way. The real movement, i.e. the surfing, is however ‘over the different parts of the underlying non temporal and non spatial reality’. This underlying reality is however structured in such away that ‘if we move through it, this influences the way time flows in case we decide to coordinate our movement in a coordinate system’.

Also with respect to the Minkowski structure of time–space and the Euclidian structure of space, we have to inverse things. First and much more primitive is the hyperbolic structure of Minkowski. It is only much afterwards that we identified a three dimensional space structure as if it could be singled out—this is most probably influenced profoundly by our local situation of very small velocities, and we are interested to analyse in great detail how this influence took shape. That such a three dimensional space structure can be considered to exist independently is not the case, and due to the error of believing that ‘places can be looked at without being influenced by their evolution in time’. ‘Time always flows for us, but also for every material entity, and that is the reason places—i.e. space—cannot be isolated without the danger of errors and we want to identify very exactly these errors. By the way, we think that a lot of confusion and difficulty in understanding relativity is due to the belief that it is linked to ‘observers’. We think that this is incorrect and that relativity describes the reality of the material entities, also when no observations at all are present. Since our bodies are material entities we are simply also with our bodies part of this relativistic reality.

## The Kinematic Reality Beneath Space–Time

Following the traditional approach it is not possible to reflect properly in the Minkowski framework, as it is never clearly stated and/or explained what the meaning is of all the four-vectors that are introduced to build the whole theory, its kinematic and its dynamics. Let us see whether our approach can shed light on these aspects. For example, what is the four-velocity?

The four-velocity $U=(u_0, u_1, u_2, u_3)$ is the derivative with respect to the intrinsic time $\tau$ of the four-position $X=(x_0, x_1, x_2, x_3)$, expressed in terms of the coordination in time–space. Let us make some calculation to see in which way the four-velocity is connected with the three-velocity. We have

28 $$\begin{aligned} u_0={dx_0 \over d\tau }={dx_0 \over dt}{dt \over d\tau }=c\, {dt \over d\tau } \end{aligned}$$

And from (2) we have

29 $$\begin{aligned} (c\, d\tau )^2=(c\, dt)^2-(dx_1)^2-(dx_2)^2-(dx_3)^2 \end{aligned}$$

so that defining

30 $$\begin{aligned} v^2= v_1^2+ v_2^2 +v_3^2 = \left( {dx_1 \over dt}\right) ^2 +\left( {dx_2 \over dt}\right) ^2+\left( {dx_3 \over dt}\right) ^2 \end{aligned}$$

and dividing (2) by $c\, dt$, we obtain:

31 $$\begin{aligned} {dt \over d\tau }={1 \over \sqrt{1-{v^2 \over c^2}}} \end{aligned}$$

From this follows that the ‘time component’ $u_0$ of the four-velocity is given by

32 $$\begin{aligned} u_0={1 \over \sqrt{1-{v^2 \over c^2}}}\, c \end{aligned}$$

For the ‘space components’ we have

33 $$\begin{aligned} u_i={dx_i \over d\tau }={dx_i \over dt}{dt \over d\tau }={1 \over \sqrt{1-{v^2 \over c^2}}}\, v_i \end{aligned}$$

When considering a single space axis, for example the $x_1$-axis, the four-velocity, with $v = v_1$, is then given by

34 $$\begin{aligned} U={1 \over \sqrt{1-{v^2 \over c^2}}}\, (c,v) \end{aligned}$$

Note that the size of this vector is

35 $$\begin{aligned} \Vert U\Vert =\sqrt{\langle U|U\rangle }=\sqrt{c^2-v^2 \over {1-{v^2 \over c^2}}}=c \end{aligned}$$

But this is the surfing velocity! So “the velocity of light in physical reality is the surfing velocity in our example of the World-Wide Web”. Of course, we need to keep taking into account the counterintuitive nature of the Minkowski metric. Although the surfing velocity is always equal to the velocity of light, the time and space components of this velocity can be very different. In fact, for a physical entity moving with constant velocity *v*, the space component is $v / \sqrt{1-{v^2 / c^2}}$, which has a magnitude between zero and infinity, increasing towards infinity with increasing value of |*v*|. This expresses that indeed for the moving entity speed goes up, equally so as time dilates and distance contracts. The time component behaves similarly, with a magnitude ranging from *c* to infinity, also increasing as |*v*| increases. In fact, *c* is obviously a limit velocity for the moving entity *B*, as measured by the not moving entity *A*. But it corresponds to an ‘infinite velocity’ of the moving entity *B* with respect to the time coordinate and an ‘infinite velocity’ of the moving entity *B* with respect to the space coordinate in the time–space frame of *A*. If we go back to our example of surfing on the World-Wide Web, we can understand it as follows, when starting from $A_0=B_0$ if we can jump without clicking any links right away to $A_m=B_l$, then in the scheme where links are counted this means ‘infinite velocity’ in all possible dimensions, be it the time or the space one. Note that the ‘possibly bigger than *c*’ intrinsic velocity of *B*’ becomes a ‘smaller than *c* not intrinsic velocity of *B* for *A*’, because there is ‘time dilation’ and ‘length contraction’ in the *A* frame, which makes hence ‘appear’ the intrinsic velocity possibly bigger than *c* for *B* as a not intrinsic velocity smaller than *c* for *A*. Hence, if we—being in the *A* frame—see something—the *B* entity—move with velocity *v* in the space realm, i.e. measuring the distance traveled per unit time in our *A* frame, then this *B* entity is actually moving with an intrinsic velocity $v / \sqrt{1-v^2/c^2}$ bigger than |*v*| and possibly also bigger than *c*. We can even easily calculate when this intrinsic velocity with magnitude bigger than |*v*| also becomes bigger than *c*. This happens when

36 $$\begin{aligned} {v \over \sqrt{1-{v^2 \over c^2}}}=c \Leftrightarrow v= \sqrt{1-{v^2 \over c^2}}c \Leftrightarrow v^2= c^2-v^2 \Leftrightarrow v= {c \over \sqrt{2}}\approx 0.7071\, c \end{aligned}$$

This shows that concerning the intrinsic velocity of a physical entity, the velocity of light is not a limit, and even not a special value to pass by. The velocity of light *c* only plays this role of ‘maximum to be reached velocity’ when the physical entity—the entity *B* in our case—is looked at from another physical entity—the entity *A* in our case.

Let us, by way of example, calculate these time and space coordinates for the example in Fig. 1. We have for this situation that

37 $$\begin{aligned} v=c\, \sqrt{{m^2-l^2 \over m^2}}=c\, \sqrt{10^2-8^2 \over 10^2}=0.6\, c=0.6 \end{aligned}$$

where for the last equality we have put $c=1$, like in Fig. 1. This means that

38 $$\begin{aligned} {1 \over \sqrt{1-{v^2 \over c^2}}}={1 \over \sqrt{0.64}}={1 \over 0.8}={5 \over 4} \end{aligned}$$

and hence the $x_1$ component of the four velocity of *B* equals

39 $$\begin{aligned} u_1=0.6 \cdot {5 \over 4}={3 \over 4} \end{aligned}$$

which is $75\%$ of the velocity of light, while *v*, the velocity of *B* measured in the time–space frame of *A* only values $60\%$ of the velocity of light.

The time component of the four velocity is

40 $$\begin{aligned} u_0={5 \over 4} \end{aligned}$$

which is $125\%$ of the velocity of light. And indeed, *B* moves more quickly from $A_0=B_0$ to $A_m=B_l$, needing 8 years, than *A* moves from $A_0=B_0$ to $A_m=B_l$, needing 10 years.

Let us calculate the size of the four-velocity. We have

41 $$\begin{aligned} \Vert U\Vert =\sqrt{u_0^2-u_1^2}=\sqrt{{25\over 16}-{9\over 16}}=\sqrt{16\over 16}=1 \end{aligned}$$

which shows that intrinsically *B*, in its own reference frame, moves with the velocity of light, like does *A* in its own reference frame. This shows well that we can interpret the velocity of light as being the ‘surfing velocity’. Indeed, also in our example of the World-Wide Web, we suppose that *A* and *B* surf with the same velocity, but *A* takes 10 click to arrive at the meeting point while *B* only takes 8 clicks.

This is in fact the moment to stand still somewhat longer with the counter intuitive aspects of the Minkowski metric, in an attempt to identify more deeply its root. Indeed, if we imagine *A* and *B* surfing with the same surfing velocity *c*, and starting together at $A_0=B_0$, then our intuition tells us that they will not arrive at the same time at $A_m=B_l$, namely *B* will arrive way before *A* arrives there. To arrive together and meet there *A* would have to surf with a higher speed. The reason for our intuition telling us this is because also for the example of the World-Wide Web we imagine it happening in a Newtonian time–space. We refer, for example, to a watch that *A* and *B* would carry along, and we want to identify the meeting place $A_m=B_l$ only as a ‘spot in space’ not linked to time. Of course this “is” how real life surfing would take place. Perhaps we would come closer to imagining an analogy if we take distance from the World-Wide Web, and think about two persons *A* and *B*, meeting in a pub $A_0=B_0$, having a specific discussion about some ‘meaning issue’, then meeting again later $A_m=B_l$ in a pub, were they discuss again. In between *A* has made 10 conceptual steps while *B* has made 8 conceptual steps. Again one could, if referring to the Newtonian world, say that *B* reflected faster than *A*. Another way is to see it as if *B* was able to take a shorter conceptual path as compared to *A* while both were all the time reflecting at the same speed. Minkowski metric and its reality teaches us that physical entities behave in the second way. Our difficulty of being able to grasp this clearly is due to our earthly bounded experience with reality, were we can mainly identify ‘spots’ in space as being the reference for ‘meetings’. That this does not bring our bodies—which are physical entities—in deep trouble, is due to our bodies being almost always at rest with respect to other physical entities. So, we have to make a specific effort with our mind to imagine the Minkowski metric related reality, and this is not a question of ‘observers related matters’. It is how realty is, and we better try to take it seriously.

For the reasons explained above it can be understood why this velocity *c* cannot be superseded when it is attempted to be put at the place of a velocity *v*, which is the velocity that a physical entity has ‘in space’. This velocity in space is quite something else than the surf velocity *c*. The velocity in space comes closer and closer to the surf velocity when the number of clicks is smaller and smaller to get from the begin webpage to the end webpage. In the time–space setting it comes to adding velocity to the space motion and subtracting velocity to the time motion, hence it comes to ‘giving an angle’ to the surf velocity. But the surf velocity itself it always equal to *c* in magnitude.

Let us go back now to the physical realm. The above insight means that material physical entities ‘move’ through the non temporal and non spatial realm of reality with a constant velocity *c*, which is equal to the velocity of light. Let us come to light itself now. Light is not a material physical entity, it has no mass, it is a boson, and not a fermion, etc. This means that the way light interacts with material physical entities is different from surfing. There are no clicks involved when moving from one page to another.

The behavior of light is a singularity of the mechanical equations of relativity. These mechanical equations are principally about the behavior of physical entities with mass, and the massless entities, like photons, are at the edge of this mechanical theory. We will see that a statement such as ‘light has velocity *c* when moving through the vacuum of the space that we constructed to give place to all physical entities’, is more subtle than imagined.

That the ‘surfing velocity’ is independent of the reference frame is natural, because it is a non temporal and non spatial velocity. Does this mean that there is an aether involved? No, because aether is a notion that only makes sense when time and space are already created and physical entities are immersed in its coordination. Unless of course aether would be conceived as a non spatiotemporal medium, which in the World-Wide Web example is precisely the Web itself. One could say that the surfing velocity being independent of the reference frame means that there is an underlying non temporal and non spatial reality, and that it is within this reality that this surfing velocity exists. It only reveals itself following our analysis when a time–space reference frame is attempted to coordinate world lines. And in the first place it reveals itself as the magnitude of the four-velocity. Only in the second place it also reveals itself as ‘how photons which do not have mass move through space to connect webpages without clicking’. Indeed, what is the intrinsic velocity in the space realm of a photon? To be able to answer this question, we need to know the four-velocity of a photon. In text books on relativity theory it is generally stated that such a velocity four-vector is not well defined, and hence that it only exists for massive physical entities. However, we can in a meaningful way consider the photon as a limit particle, and hence also calculate its four-velocity as the four-vector obtained in this limit. We find then $\infty$ for the time component and also $\infty$ for each of the space components. But its size is equal to *c* as is the case for a massive physical entity. A photon travels with surfing velocity equal to *c* in the non temporal and non spatial realm, but in its own time–space frame it does not need a click to move in the time direction, nor it needs to run through length units in the space realm. This is the Minkowsky nature of physical reality in its weirdest aspect. Indeed, when looked at the photon from any other frame, it moves exactly with velocity *c* in the time–space of this other frame. It is the ‘any other’ which is at the origin of the constancy of this velocity of the photon in any reference frame. But, let us remember that also this velocity *c*, exactly like any velocity *v* of any massive physical entity, is ‘not’ an intrinsic property of the entity in question. It is a property changed due to it being looked at from another reference. This is equally so for a photon, the velocity *c* is ‘not’ its intrinsic space realm velocity, because this one is infinite.

## The Dynamic Reality Beneath Space–Time

What about the relativistic dynamics? Often it is said that when the velocity *v* increases the mass increases and that this is the reason why a physical entity cannot reach the velocity of light, its mass going to infinity with increasing velocity. We believe that this is a wrong way of looking at the dynamical aspects of the situation. Indeed, remember that the space component of the four-velocity—reasoning here, for simplicity, with only one space dimension—is given by

42 $$\begin{aligned} u={1 \over \sqrt{1-{v^2 \over c^2}}}\, v \end{aligned}$$

which means that this component goes to infinity when |*v*| goes to *c*. Of course, also the time component of the four velocity being equal to

43 $$\begin{aligned} u_0={1 \over \sqrt{1-{v^2 \over c^2}}}\, c \end{aligned}$$

goes to infinity when |*v*| goes to *c*. That the magnitude of the four-velocity remains finite, and a constant equal to *c*, is linked to the special form of the Minkovski metric, which subtracts two infinities, which of course can lead to a finite quantity. All this means that already on the level of the kinematics the behavior of light—moving through space with velocity *c*—is singular. Mass however does not behave singularly at all in our opinion, rather, it is an invariant, hence an intrinsic property of the physical entity under consideration. Of course, this is well known, and acknowledged in standard textbooks of relativity, for what concerns the ‘rest mass’. We believe that it is the only mass that exists, the one that is measured when a physical entity is at rest with respect to the reference frame where the mass measurement is carried out. The apparent increase of mass with velocity is due to the increase of the spatial coordinate of the four-velocity with velocity. Hence, we believe that one should not speak about ‘relativistic mass’, like in many textbooks of relativity theory.

The four-momentum *P* of a massive physical entity—in the absence of a magnetic field—is the mass multiplied by the four-velocity *U*, hence

44 $$\begin{aligned} P=m\, U \end{aligned}$$

Let us note right away that we can calculate the magnitude of the four-momentum, and then get

45 $$\begin{aligned} \Vert P\Vert =\sqrt{\langle P|P\rangle } =m\sqrt{\langle U|U\rangle }=m\, c \end{aligned}$$

The size of the four-momentum of a massive physical entity is also an invariant, and it is the momentum of a mass *m* with velocity *c*. In our example, it is the surfing momentum. What in our example could be the equivalent of mass? We think it is ‘meaning impact’, i.e. the size with which the meaning impacts—we use even in every day language the expression ‘impact’ when it concerns meaning. Of course, this again only makes sense in case we see surfing not just as a passive action with respect to fixed webpages, but as a dynamical action, were every visit of a webpage also potentially changes the meaning content of this webpage. When two massive physical entities collide, the equivalent would be that two webpages come into competition, both wanting to occupy the same state. If both are solids—webpages that are very stubborn in adapting and/or making compromise in meaning content with each other—the collision can be of the elastic type. But collisions can also lead to merging giving rise to a third webpage containing a consensus of the two colliding ones, which would correspond to an inelastic collision, with possible creation of entanglement.

From (45), we find the formula of relativity, expressing energy in function of mass. That, of course, is also linked to interpreting the time component of the four-momentum as ‘the energy divided by the velocity of light’, hence

46 $$\begin{aligned} P=\left( {E \over c}, p_1, p_2, p_3\right) \end{aligned}$$

so that we have

47 $$\begin{aligned} \langle P|P\rangle ={E^2 \over c^2}-p_1^2-p_2^2-p_3^2=m^2c^2 \end{aligned}$$

If we consider the situation of a physical entity at rest, which means that $p_1=p_2=p_3=0$, we get

48 $$\begin{aligned} E=m c^2 \end{aligned}$$

which is the famous formula derived by Albert Einstein in his 1905 work on the theory of special relativity (Einstein 1905b). Let us remark that a part of the energy comes from the momentum *mc* that any mass carries with itself in its ‘flow in time’—and taking into account the existence of the non temporal underlying reality, we should say ‘the momentum that the mass, i.e the meaning impact, carries while moving through the non temporal reality, moving through the overall meaning structure’, and that this appears like ‘moving in time’ is due to the time–space coordination creation of the situation. So, instead of interpreting mass-energy as potential energy, we can now interpret it as kinetic energy. If mass is turned into light energy, this is as ‘taking away the huge momentum that the mass has in its flow through time—its motion of surfing’ and giving it away in the form of light.

What about light? Since in standard relativity textbooks one considers that no meaning can be given to the four-velocity for light, also the formula $P=mU$ is not considered valid for light. However, in our case, where we have considered *U* to exist for light, we can investigate whether we can keep $P=mU$ as valid for light too. Let us first consider the value of the four-momentum. For $m=0$ we have

49 $$\begin{aligned} \langle P|P\rangle ={E^2 \over c^2}-p_1^2-p_2^2-p_3^2=0 \end{aligned}$$

which leads to

50 $$\begin{aligned} E=c\, p \end{aligned}$$

where *p* is the magnitude of the three-momentum, and hence

51 $$\begin{aligned} P=(p, p_1, p_2, p_3) \end{aligned}$$

which shows that *P* is a null four vector, located on the light cone, which we would expect for light. The sizes of *p*, $p_1$, $p_2$ and $p_3$ are determined by quantum theory as a consequence of Planck’s law $E=h\nu$ and De Broglie’s equations, hence

52 $$\begin{aligned} p={h\nu \over c} \quad p_1=2\pi h k_1 \quad p_2=2\pi h k_2 \quad p_3=2\pi h k_3 \end{aligned}$$

where *h* is Planck’s constant and $(k_1, k_2, k_3)$ is the wave vector of the photon. Of course, strictly speaking we leave the framework of relativity theory if we use these quantum formula’s to define the four-momentum of a photon. We elaborate further on relativity and quantum in Sect. 8.

Let us remark explicitly that Fig. 1, since it is drawn in a (*t*, *x*)-reference frame, might give the impression that the points of this place are points of a Euclidean plane. This is another—although related to the one we mentioned before—aspect that makes the Minkowski metric so counter intuitive. The path taken by *B*, if we look at the (*t*, *x*)-frame as an Euclidean plane obviously is much longer than the path taken by *A*. While following the Minkowski metric it is the other way around. Light which takes a path inclined $45^\circ$ is the shortest possible path, while in an Euclidean view of the plane used in Fig. 1 this is even longer than both paths, the one taken by *A* and the one taken by *B*. Our minds, when they see a plane, can imagine this plane to be Euclidean. We can imagine a curvature that deviates in only a smooth way from the Euclidean situation, since we know what twisted pieces of paper are, for example. But Minkowski really deviates profoundly from Euclide, so our imagination fails about it. However, experiments confirming abundantly the theory of relativity show us that ‘this is the way reality is’. When space is explored starting from time and an underlying non temporal and non spatial reality, in the way we analyzed above, then the plane depicting a time and a space coordinate is Minkowskian and not Euclidean. This however is ‘not’ due to observation, hence the traditional way that relativity is attempted to be explained obscures its essence in our opinion. The Minkowski structure arises from deep reality itself, and is explored by physical entities with mass and photons of light. Human beings and more even human observers do not play any special role in it. However, the reason we find the Minkowski metric so counter intuitive when we see it exposed, and more even when we see it drawn on a plane, is deeply rooted in the nature of human observation. We are used to see paths in space only without involving the effect that velocity has on reaching into the future. The reason is that we and all customary physical entities around us move ‘in space’ extremely slowly—remember that every physical entity moves with the velocity of light in time–space, or, more correctly, in the non temporal and non spatial underlying reality.

Light does play a crucial role in our experience too. But light, because it consists of physical entities with zero mass, cannot be ‘joined by us’. We, bound to our physical bodies which have masses different from zero, when we want to introduce a time–space frame for ourselves, ‘cannot do this making it the time–space frame were light is in’. This means that we can only observe light from the outside, in our time–space frame, and not in its own. That is the reason that light shows itself to us in the way we perceive it, namely ‘always’ moving with a space—in our time–space frame—velocity equal to *c*, exactly the same quantity with which we move in our own time–space frame in the direction of our four-velocity vector, which means, partly in our time direction, and partly in our space direction—were also we see light moving.

Light moves so quickly ‘in the space realm of any time–space frame from where we observe it’, namely with speed *c* that we hardly see it moving with the senses that we have acquired from our biological evolution in our space realm, so we are only confronted with other properties of light than the ones that could us make aware of the Minkowskian nature of reality directly. Indirectly it is light that showed us the way, first in its appearance in the form of electricity and magnetism, leading to Maxwell’s equations. Light might also carry some of the other keys for the deeper reasons of the exact structure of space, i.e. Euclidean, since it is so much present in it.

Can we understand more profoundly how our Euclidean intuition has grown and misguided us in our image about time–space? We indeed can. Space as a distinct entity with three dimensions arose as a consequence of a further exploration of the time–space in the way we analyzed above. Till now we have neglected to make a difference between a situation were there would be more than one dimension of ‘space’ and a situation with only one space dimension. But, the Minkowski metric of the experienced mechanics and electromagnetism of our world gives rise to a three-dimensional space. It is, in fact, also this Euclidian nature of the three-dimensional space which makes it so difficult for us to intuitively imagine the nature of hyperbolic time–space. How are we confronted with this more than one dimension of space and its Euclidean nature? Let us investigate this question.

## The Reality of Three-Dimensional Space

If we consider only two world lines elements $(A_n)_n$ and $(B_k)_k$ of ${{\mathcal {A}}}_{0,m}$, like we did so far, we will not need more than one space dimension to fit them in the Minkowski structure. Hence, let us consider a third world line $(C_i)_i=\{C_0, \ldots , C_j\}$ of a physical entity *C*, element of ${{\mathcal {A}}}_{0,m}$. This means that we have $A_0=B_0=C_0$ and $A_m=B_l=C_j$. Without loss of generality we can suppose that $j \le l \le m$ which means that $(C_i)_i$ is the fastest of the three considered paths in heading towards the reunion of the three. Let us analyse what happens with the different velocities that come into play as a consequence of the Minkowski metric. The velocity $v^{A}_{C}$ with which *C* moves in the time–space coordination of *A* is given by

53 $$\begin{aligned} v^{C}_{A}=c\, \sqrt{m^2-j^2 \over m^2} \end{aligned}$$

which is bigger than the velocity $v^{B}_{A}$ with which *B* moves in the time–space coordination of *A*

54 $$\begin{aligned} v^{B}_{A}=c\, \sqrt{m^2-l^2 \over m^2} \end{aligned}$$

because $j \le l$. The first question we want to consider is ‘whether the nature of the Minkowski coordination of our non temporal and non spatial reality’ indicates the existence of at least such a *C*, different from *A* and *B*. The answer is ‘yes’, but we will also consider the situation where $j=l$, i.e. where the time needed to reach the meeting point $A_m=B_l=C_j$ for the two paths *B* and *C* is the same. And even in this more simple situation we will see that the Minkowski structure induces more than one space dimension. Let us consider for a moment the situation of Fig. 1, but now with *B* and *C* moving in opposite direction. Then, already for this situation, Minkowski allows for $(C_i)_i$ to be a different path than $(B_k)_k$, although both paths are located in one and the same space direction in this case, which means that by means of this case no extra space dimension is yet induced by the Minkowski structure. For this we will have to consider a situation were $(C_i)_i$ moves in another direction, not just the opposite of the direction in which $(B_k)_k$ moves.

We start our investigation by considering the Lorentz transformation that connects the coordinates of a four-vector in the *B* time–space coordination with the coordinates of the four-vector in the *A* time–space coordination. This Lorentz transformation is given by

55 $$\begin{aligned} L(B,A)=\left( \begin{array}{cccc} {1 \over \sqrt{1-\left( {v^{B}_{A} \over c}\right) ^2}} &{} {-{v^{B}_{A} \over c} \over \sqrt{1-\left( {v^{B}_{A} \over c}\right) ^2}} &{} 0 &{} 0 \\ {-{v^{B}_{A} \over c} \over \sqrt{1-\left( {v^{B}_{A} \over c}\right) ^2}} &{} {1 \over \sqrt{1-\left( {v^{B}_{A} \over c}\right) ^2}} &{} 0 &{} 0 \\ 0 &{} 0 &{} 1 &{} 0 \\ 0 &{} 0 &{} 0 &{} 1 \end{array} \right) \end{aligned}$$

**Fig. 2:**
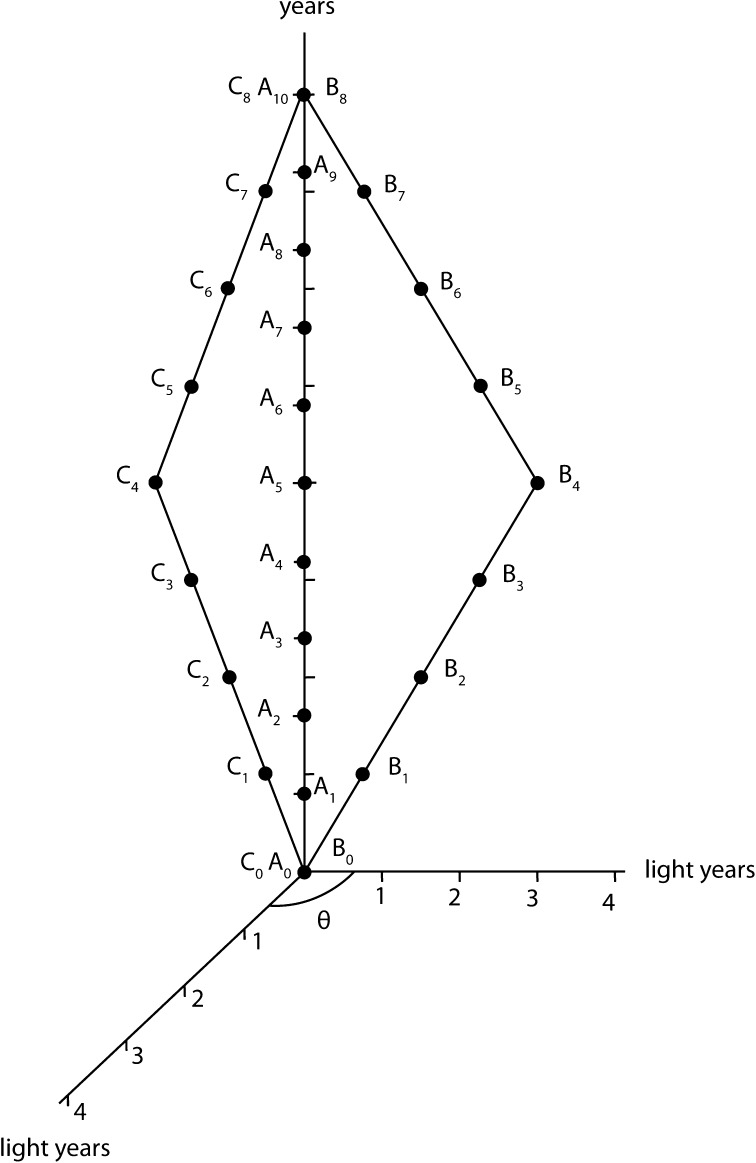
A graphical representation of *A*, *B* and *C* for $m=10$ and $l=j=8$

Let us now consider the situation of Fig. 1, hence for the $(A_n)_n$ world line we have $m=10$ and for the $(B_k)_k$ world line we have $l=8$. We consider $(C_i)_i$ with $j=l=8$. We make the hypothesis that for *B* a space dimension $x_1$ comes into being coordinating the more speedy motion of *B* towards the meeting point with *A* at $A_{10}=B_{8}$. And now we add the hypothesis that for *C* another space dimension $x_2$ comes into being coordinating the more speedy motion of *C* towards the meeting point at $A_{10}=B_{8}=C_{8}$. We have represented this situation in Fig. 2. Let us write down the four-velocity for *A*, *B* and *C* in the *A* time–space frame that expresses the above hypothesis. We have

56 $$\begin{aligned} U^{A}_{A}= & {} {1 \over \sqrt{1-\left( {v^{A}_{A} \over c}\right) ^2}}(c, 0, 0, 0) \end{aligned}$$

57 $$\begin{aligned} U^{B}_{A}= & {} {1 \over \sqrt{1-\left( {v^{B}_{A} \over c}\right) ^2}}\left( c, v^{B}_{A}, 0, 0\right) \end{aligned}$$

58 $$\begin{aligned} U^{C}_{A}= & {} {1 \over \sqrt{1-\left( {v^{C}_{A} \over c}\right) ^2}}\left( c, v^{C}_{A}\cos \theta , v^{C}_{A}\sin \theta , 0\right) \end{aligned}$$

where $v^{A}_{A}$, $v^{B}_{A}$ and $v^{C}_{A}$ are the sizes of the ‘space velocities’ related to *A*, *B* and *C*, and $\theta$ is the angle which parametrizes the direction of the space velocity related to *C* in the $(x_1x_2)$-plane. So, similarly to the situation considered in Fig. 1, for the added *C*, we have

59 $$\begin{aligned} v^{A}_{A}=0 \quad v^{B}_{A}=v^{C}_{A}=0.6\, c \quad \theta \in [0, 2\pi ] \end{aligned}$$

This gives

60 $$\begin{aligned} U^{A}_{A}= & {} (c, 0, 0, 0) \end{aligned}$$

61 $$\begin{aligned} U^{B}_{A}= & {} {5 \over 4}\left( c, {3 \over 5}, 0, 0\right) \end{aligned}$$

62 $$\begin{aligned} U^{C}_{A}= & {} {5 \over 4}\left( c, {3 \over 5}\cos \theta , {3 \over 5}\sin \theta , 0\right) \end{aligned}$$

63 $$\begin{aligned} L(B,A)= & {} \left( \begin{array}{cccc} {5 \over 4} &{} -{3 \over 4} &{} 0 &{} 0 \\ -{3 \over 4} &{} {5 \over 4} &{} 0 &{} 0 \\ 0 &{} 0 &{} 1 &{} 0 \\ 0 &{} 0 &{} 0 &{} 1 \end{array} \right) \end{aligned}$$

We can calculate the four-vectors in the *B* time–space frame. This gives

64 $$\begin{aligned} U^{A}_{B}& {}= L(B,A)U^{A}_{A} \end{aligned}$$

65 $$\begin{aligned} U^{B}_{B}& {}= L(B,A)U^{B}_{A} \end{aligned}$$

66 $$\begin{aligned} U^{C}_{B}& {}= L(B,A)U^{C}_{A} \end{aligned}$$

and hence

67 $$\begin{aligned} U^{A}_{B}= & {} \left( \begin{array}{c} {u^{A}_{B}}_0 \\ {u^{A}_{B}}_1 \\ {u^{A}_{B}}_2 \\ {u^{A}_{B}}_3 \end{array} \right) =\left( \begin{array}{cccc} {5 \over 4} &{} -{3 \over 4} &{} 0 &{} 0 \\ -{3 \over 4} &{} {5 \over 4} &{} 0 &{} 0 \\ 0 &{} 0 &{} 1 &{} 0 \\ 0 &{} 0 &{} 0 &{} 1 \end{array} \right) \left( \begin{array}{c} c \\ 0 \\ 0 \\ 0 \end{array} \right) = \left( \begin{array}{c} {5 \over 4} c \\ -{3 \over 4} c \\ 0 \\ 0 \end{array} \right) \end{aligned}$$

68 $$\begin{aligned} U^{B}_{B}= & {} \left( \begin{array}{c} {u^{B}_{B}}_0 \\ {u^{B}_{B}}_1 \\ {u^{B}_{B}}_2 \\ {u^{B}_{B}}_3 \end{array} \right) =\left( \begin{array}{cccc} {5 \over 4} &{} -{3 \over 4} &{} 0 &{} 0 \\ -{3 \over 4} &{} {5 \over 4} &{} 0 &{} 0 \\ 0 &{} 0 &{} 1 &{} 0 \\ 0 &{} 0 &{} 0 &{} 1 \end{array} \right) \left( \begin{array}{c} {5 \over 4} c \\ {3 \over 4} c \\ 0 \\ 0 \end{array} \right) = \left( \begin{array}{c} {25 \over 16} c-{9 \over 16} c \\ -{15 \over 16} c + {15 \over 16} c \\ 0 \\ 0 \end{array} \right) \end{aligned}$$

69 $$\begin{aligned} U^{C}_{B}= & {} \left( \begin{array}{c} {u^{C}_{B}}_0 \\ {u^{C}_{B}}_1 \\ {u^{C}_{B}}_2 \\ {u^{C}_{B}}_3 \end{array} \right) =\left( \begin{array}{cccc} {5 \over 4} &{} -{3 \over 4} &{} 0 &{} 0 \\ -{3 \over 4} &{} {5 \over 4} &{} 0 &{} 0 \\ 0 &{} 0 &{} 1 &{} 0 \\ 0 &{} 0 &{} 0 &{} 1 \end{array} \right) \left( \begin{array}{c} {5 \over 4} c \\ {3 \over 4} c \cos \theta \\ {3 \over 4} c \sin \theta \\ 0 \end{array} \right) = \left( \begin{array}{c} {25 \over 16} c-{9 \over 16} c \cos \theta \\ -{15 \over 16} c + {15 \over 16} c \cos \theta \\ {3 \over 4} c \sin \theta \\ 0 \end{array} \right) \end{aligned}$$

Hence we find

70 $$\begin{aligned} U^{A}_{B}& {}= \left( {5 \over 4}c, -{3 \over 4}c, 0, 0\right) \end{aligned}$$

71 $$\begin{aligned} U^{B}_{B}& {}= (c, 0, 0, 0) \end{aligned}$$

72 $$\begin{aligned} U^{C}_{B}= & {} \left( c+{9 \over 16} c(1- \cos \theta ) , -{15 \over 16} c (1 - \cos \theta ), {3 \over 4} c \sin \theta , 0\right) \end{aligned}$$

which makes it possible to calculate the space velocities in the *B* time–space frame. We find

73 $$\begin{aligned} {\vec {v}}^{A}_{B}=\left( {{u^{A}_{B}}_1 \over {u^{A}_{B}}_0},{{u^{A}_{B}}_2 \over {u^{A}_{B}}_0},{{u^{A}_{B}}_3 \over {u^{A}_{B}}_0}\right) c=\left( -{3 \over 5}\, c, 0, 0\right) =\left( -0.6\, c, 0, 0\right) \end{aligned}$$

This is what we expect. If *B* moves with space velocity $0.6\, c$ in the positive $x_1$-direction in the *A* time–space frame, then *A* moves in the *B* time–space frame with the same velocity in the opposite direction, hence in the negative $x_1$-direction. We also find

74 $$\begin{aligned} {\vec {v}}^{B}_{B}=\left( {{u^{B}_{B}}_1 \over {u^{B}_{B}}_0},{{u^{B}_{B}}_2 \over {u^{B}_{B}}_0},{{u^{B}_{B}}_3 \over {u^{B}_{B}}_0}\right) c=(0, 0, 0) \end{aligned}$$

This is also what we expect. In the *B* time–space frame *B* is at rest. And we also find

75 $$\begin{aligned} {\vec {v}}^{C}_{B}= & {} \left( {{u^{C}_{B}}_1 \over {u^{C}_{B}}_0},{{u^{C}_{B}}_2 \over {u^{C}_{B}}_0},{{u^{C}_{B}}_3 \over {u^{C}_{B}}_0}\right) c=\left( {-{15 \over 16} c (1 - \cos \theta ) \over c+{9 \over 16} c(1- \cos \theta )}, {{3 \over 4} c \sin \theta \over c+{9 \over 16} c(1- \cos \theta )}, 0\right) c \end{aligned}$$

76 $$\begin{aligned}= & {} \left( -{{15 \over 8}c\sin ^2{\theta \over 2} \over 1+{9 \over 8}\sin ^2{\theta \over 2}}, {{3 \over 2}c\sin {\theta \over 2}\cos {\theta \over 2} \over 1+{9 \over 8}\sin ^2{\theta \over 2}}, 0\right) = {3 \over 2}c\sin {\theta \over 2}\left( -{{5 \over 4}\sin {\theta \over 2} \over 1+{9 \over 8}\sin ^2{\theta \over 2}},{\cos {\theta \over 2} \over 1+{9 \over 8}\sin ^2{\theta \over 2}}, 0\right) \end{aligned}$$

What is now very interesting is that the magnitude of ${\vec {v}}^{C}_{B}$, hence $\Vert {\vec {v}}^{C}_{B}\Vert$, is no longer constant, and changes with $\theta$. This means that we can ‘detect’ the presence of a second dimension by purely measuring the magnitude of the space velocity in the *B* time–space frame. The reason is very deep, namely that the Lorentz transformation only provokes a length contraction in the direction of the space velocity of the moving reference frame, and not in the other directions. Let us calculate the size $\Vert {\vec {v}}^{C}_{B}\Vert$ of ${\vec {v}}^{C}_{B}$. We get

77 $$\begin{aligned} \Vert {\vec {v}}^{C}_{B}(\theta )\Vert =\sqrt{\left( {v^{C}_{B}}_1\right) ^2+\left( {v^{C}_{B}}_2\right) ^2}={3 \over 2}c\sin {\theta \over 2}\sqrt{\left( {{5 \over 4}\sin {\theta \over 2} \over 1+{9 \over 8}\sin ^2{\theta \over 2}}\right) ^2+\left( {\cos {\theta \over 2} \over 1+{9 \over 8}\sin ^2{\theta \over 2}}\right) ^2}. \end{aligned}$$

For the specific values $\theta =0$, $\theta =\pi$ and $\theta ={\pi \over 2}$ a simple calculation gives

78 $$\begin{aligned} \Vert {\vec {v}}^{C}_{B}(0)\Vert =0\quad \Vert {\vec {v}}^{C}_{B}(\pi )\Vert =0.88c\quad \Vert {\vec {v}}^{C}_{B}\left( {\pi \over 2}\right) \Vert =0.77c \end{aligned}$$

**Fig. 3:**
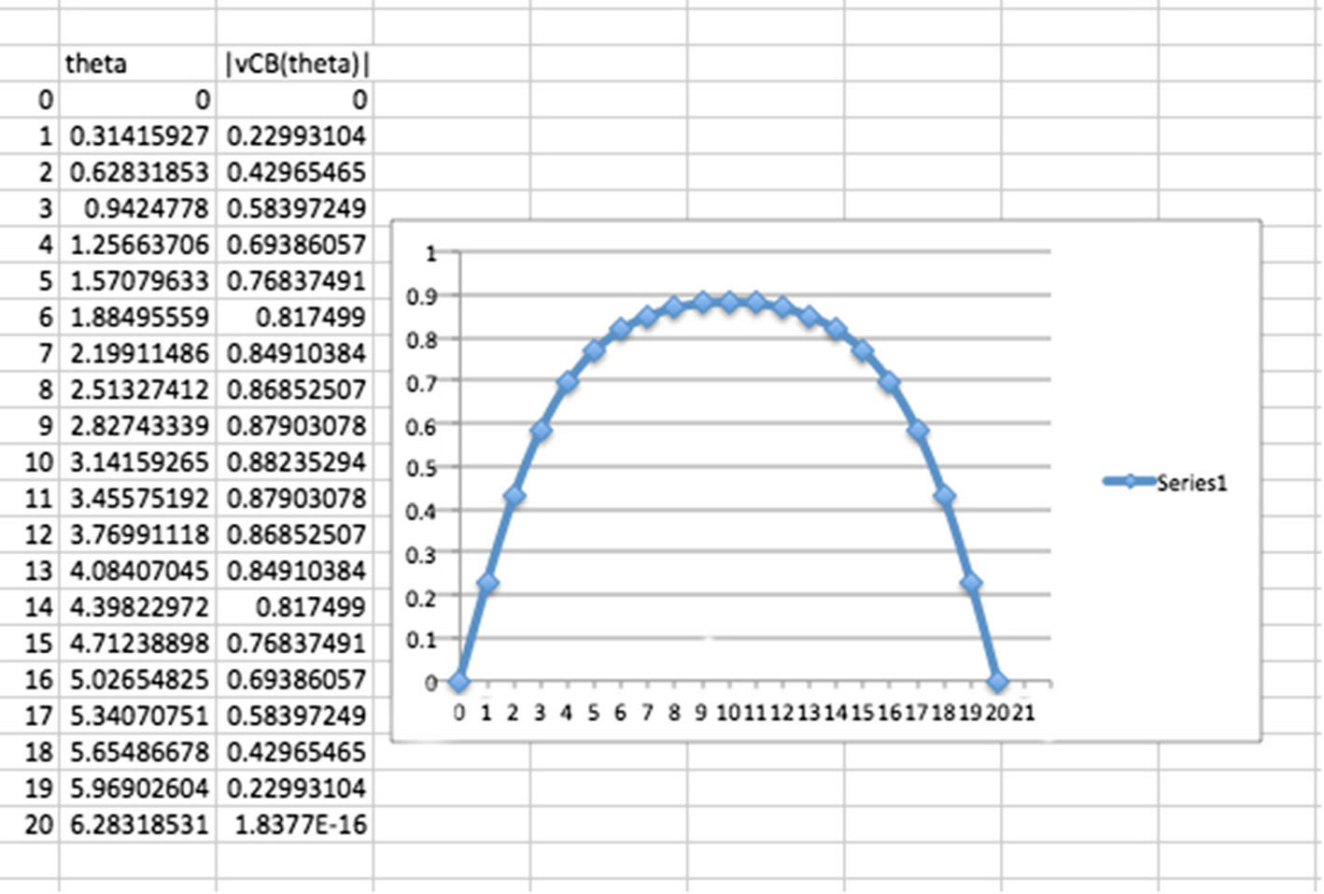
A graphical representation of $\Vert {\vec {v}}^{C}_{B}(\theta )\Vert$ for different values of $\theta$

Hence, for a velocity ${\vec {v}}^{C}_{A}$ equal in size, namely $0.6\, c$, and in the same direction as ${\vec {v}}^{B}_{A}$ in the *A* time–space frame, which is what the value $\theta = 0$ represents, we get a velocity $\Vert {\vec {v}}^{C}_{B}(0)\Vert =0$ in the *B* time–space frame. This is what we would expect, and we considered already this case above. For a velocity ${\vec {v}}^{C}_{A}$ equal in size, namely $0.6\, c$, but in opposite direction with respect to ${\vec {v}}^{B}_{A}$ in the *A* time–space frame, which is what the value $\theta = \pi$ represents, we get a velocity $\Vert {\vec {v}}^{C}_{B}(0)\Vert =0.88\, c$. This situation would still not need an extra space dimension, and it also correspond to what we might expect from the well-known sum-of-relativistic-velocity formula. Here comes an interesting new type case. For a velocity ${\vec {v}}^{C}_{A}$ equal in size, namely $0.6\, c$, but in a direction orthogonal to ${\vec {v}}^{B}_{A}$ in the *A* time–space frame, which is what the value $\theta = {\pi \over 2}$ represents, we get a velocity $\Vert {\vec {v}}^{C}_{B}(0)\Vert =0.77\, c$. This ‘size of space velocity’ in the *B* time–space frame cannot be explained without the introduction of more than one space dimension. In Fig. 3, we have represented the graph of the sizes of the space velocity $\Vert {\vec {v}}^{C}_{B}(\theta )\Vert$ for different values of $\theta$. The scale of the $\theta$ variable is divided in 20, which means $18^\circ$ for each unit. We can see that $\Vert {\vec {v}}^{C}_{B}(\theta )\Vert$ increases from 0 to ${15 \over 17}\, c=0.88\, c$ when $\theta$ varies from 0 to $\pi$, and then decreases from ${15 \over 17}\, c=0.88\, c$ to 0 when $\theta$ varies from $\pi$ to $2\pi$ . All the intermediate values can be measured in the *B* time–space frame ‘as sizes of the space velocity of *C*’. This is a straight forward indication of the ‘necessity to introduce more than one space dimension’ if coping with the ‘facts of reality and the data about sizes of space velocities extracted from it’ that we can move with different four-velocities towards meeting points. Or, if we go back to our surf and World-Wide Web example, we need more than one space dimension if we want to cope with ‘space and space velocities’ as remedies for the fact that we can reach a meeting point by different number of clicks, and in such a way that (i) there is a ‘slowest path’, i.e. a path needing the higher number of clicks, and (ii) there are multitudes of faster paths that are different. Of course, we should not forget that when we reason on the World-Wide Web we know from our daily experience, things take place in an Euclidian setting, while the time–space realm is Minkowskian. This means that the words ‘slowest’ and ‘fastest’ should be exchanged when we want to use our Euclidian intuition for a Minkowskian World-Wide Web equivalence.

## Quantum and Relativity

We have mentioned that our analysis is inspired by what we have learned in the context of quantum theory. We have indeed focused ourselves on the identification of a reality based on the intrinsic (proper) elements which can be identified in relativity theory. This is methodologically equivalent to how we have contributed in the past to the elaboration of an operational-realistic approach to quantum theory (Aerts 1982, 1983) and our more recent contextuality interpretation and explanation of quantum theory proceeds also along this method rationale (Aerts 2009, 2010a, b, 2013, 2014). Also, the new foundation of relativity theory we have presented here can be considered as being part of our general endaveour of building realistic interpretations, and since we use explicitly the analogy of the World-Wide Web one might think of the non temporal and non spatial deep reality to be conceptual in nature. This however is not a necessity for the core aspects of our analysis to remain true, but in case we would add it as an extra hypothesis this new foundation of relativity theory would become part of our general contextuality interpretation and explanation of quantum theory.

So far we have only considered macroscopic physical entities which are fairly separated from each other and light, without giving any thought to how typical quantum entities would behave in terms of relativity. Quite some successful theories have been worked out that are quantum and relativistic, but including gravity has failed till now, i.e. quantum and gravity have not yet been able to be reconciled into a common theory. In this section, we will give an outline of ideas and of their aspects that we want to investigate further.

One of the great sucess stories of quantum and relativity is that of the Dirac equation for the electron (Dirac 1928). With exceptional ingenuity, Dirac constructed a wave equation of the first order in time and space—i.e. handling time and space on the same footing, as opposed to Schrödinger’s equation, which is first order in time and second order in space—leading to a ‘squared’ version compatible with the well-known second order in time and second order in space ‘wave equations’ of classical electrodynamics. In this way, the spin of the electron spontaneously made its appearance, additionally introducing two new components, which led to the theoretical description of what turned out to be anti-matter. It is known that anti-matter can also be looked upon as matter ‘moving in the opposite direction in time’.

If matter ‘moves’ at a velocity *c*, by surfing over a non spatial and non temporal reality in a specific direction, it is not difficult to imagine that it will also be possible for matter to surf in the opposite direction. But matter which surfs backward will reveal itself as anti-matter when looked upon from a reference frame connected with matter surfing forward. The same connection of time–space creation with a surfing movement above a non temporal and non spatial reality can explain the time-inversion symmetry of electromagnetic laws.

That relativity theory is also valid for quantum particles and even for the most fundamental ones, is an uncontestable experimental fact. Indeed, the lifetimes of elementary particles measured in particle colliders show in a manifest way the effect of time dilation. In this sense we wonder whether our view on relativity theory and more generally our contextuality interpretation could explain why our universe consists predominantly of matter with little presence of anti-matter while on the level of fundamental quantum theory matter and anti-matter are symmetric in their presence. That the view we expressed here on relativity theory can be seen as a part of our contextuality interpretation (Aerts 2009, 2010a, b, 2013, 2014) means that we consider the fundamental interaction on the quantum level as being of a conceptual nature, i.e. quantum particles are conceptual entities that mediate by means of a proto-language between pieces of matter. This also means that there is a fundamental evolutionary aspect present in the micro-realm comparable to the evolutionary aspect which is present in human culture. It is plausible that this evolution was taking place from the very early stages of our universe. This means that the question ‘why is our universe predominantly made of matter, with little presence of anti-matter’ might have an evolutionary answer which is ‘that only in this way matter could have evolved to what it is now’. Or, with other words, matter has separated from anti-matter because it was the only way to evolve and remain in existence. That both surf in opposite directions with the velocity of light over a non temporal and non spatial reality is a more concrete indication of this evolutionary mechanism. But, separation from anti-matter was not the only obstacle for matter to secure its existence. And here we come to what we believe to be a fundamental role of gravity and mass and hence of the general theory of relativity.

Remember that in our analysis we identified the deep structure of relativity theory in the hyperbolic nature of the time–space relation. The Euclidian ‘space realm’ within a specific time–space frame only plays the role to allow a second physical entity *B* to move in such a way that it can ‘meet again’ with a first physical entity *A*. This ‘meeting again’ is only possible after *B* has made a ‘turn back’ towards *A* after first speeding away from *A*. If such a ‘turn back’ would not be possible *A* and *B* would only meet once and never meet again, and hence the mere reason to even consider a time–space frame would disappear. The physical universe would be a universe without any form of consistency, because different entities would never meet more than once. This also would mean that no two different entities would even be able to ‘remain together’, except if they would be ‘at rest’ forever with respect to each other, which would be a reality lacking fundamental aspects with respect to the reality we know. In the situation we considered in Sect. 2 we have been reasoning as if the physical entity *B* can easily ‘turn back’ towards the physical entity *A*, and intuitively, because these are the examples usually chosen in relativity theory, we think of a space ship steered by humans and hence being able to turn back in the way space ships can do. But that is only a very recent possibility for the ‘turning back’ of a physical entity *B* towards another physical entity *A* to secure a second meeting. This is effectively also the way we personally return home by car, train, bike or on foot in the evening after having gone to work in the morning and hence make it possible to meet again our home. Before human culture came into existence this way to ‘return and meet again’ and more generally ‘to stay together’ did not exist, but there were and still are the forces and the way they exert their influence. Remember that in our contextuality interpretation these forces are one side of the evolutionary dynamics, namely the side where the different proto-languages exist, mediating between the entities that are influenced by these forces.

Let us first consider the weakest of all these forces, gravity. Gravity works in duality with mass, also in general theory of relativity. And again the hypothesis that an evolutionary aspect is at work is not implausible. Definitely on the level of macroscopic physical entities, before cultures like the human one could navigate cars, trains, bicycles and space ships and hence organise a ‘meeting again’ and a ‘staying together’ as a consequence of the cultural force carried by language and communication, it was and still is mainly gravity which organises the ‘meeting again’ and ‘staying together’ of macroscopic physical entities. More concretely, if the solar system remains a whole, where the ‘meeting again’ and ‘staying together’ is organised by light reflecting between the different planets, this is because the masses of sun and planets curve time–space in such a way that all of the collaborating physical entities moving on the geodesics of this curved time–space—having the hyperbolic Minkowsky structure when gravity tends to zero—surf with the velocity of light on these geodesics and each of them individually can frame the others within a curved time–space structure where the curved space realm—having the Euclidian structure when gravity tends to zero—is only needed to cope with the time dilations of the others. It remains true for situations described by mass and how it curves time–space that all the others move faster than the one doing the framing in the time of the latter. The curvature makes that this is true for all.

The situation for the matter that clumps together to form stars and planets is slightly different. Also the ‘meeting again’ and ‘staying together’ is organised by gravity and its communication with mass, but it does not happen on geodesics. The reason is that the matter is stopped in its geodesic free fall towards the center of the star or the planet in a type of ‘matter traffic jam’, except when the entity in question is not a planet or a star but a black hole, i.e. when the distance from the center where the matter traffic jam stops is smaller than the Schwarzschild radius. Since the matter of stars and planets is stopped in their geodesic free fall, it is not moving on geodesics, but undergoes an outward acceleration by being pushed back at falling towards the center of the star or planet—the ‘pushing back’ being the result of other non-gravitational forces, and quantum effects like the Pauli exclusion principle, i.e. degeneracy pressure. But the ‘staying together’ is secured.

On the level of atoms and molecules it is the electromagnetic force which organises the ‘meeting again’ and the ‘staying together’. The mediators are the photons of light and we have a relativistic quantum theory, namely Quantum Electrodynamics, which describes to a very high degree of accuracy what happens at this scale. The realm where atoms and molecules, and also sub atomic particles, exist, although relativity theory is definitely still valid in this realm, is so strongly ‘quantum’, that time–space in the way we know it with respect to macroscopic physical entities no longer makes sense. We cannot meaningfully speak of a ‘quantum entity being inside space’ as we can do for a macroscopic physical entity. On the other hand, is not the lesson from our approach to relativity theory exactly that ‘we should not see space in this way, i.e. like a container of entities?’. Indeed, and let us reflect now about quantum entities with this new attitude, of the space realm being only introduced to cope with the different time evolutions of the different considered entities’. We start by noting that the states of a quantum entity—not considering for simplicity their spin—are elements of $L^2({\mathbb {R}}^3)$, the collection of complex square integrable functions of three real variables, i.e. the so called wave functions. The connection to three-dimensional Euclidian space is these three real variables which represent the locations where the quantum entity can be found when a measurement to detect it is executed and the change of state happening during such a measurement is called a collapse. The collapsed states, i.e. the states after a measurement of position, could possibly still be looked at as being states of the entity ‘inside space’, but this is definitely not the case for the other states, which are the majority. We introduced the notion of ‘non spatiality’ to indicate the nature of the non collapsed states of a quantum entity. This situation is still compatible with the example of the World-Wide Web that we introduced and more generally with the contextuality interpretation of quantum theory. Indeed, when a website is looked at, and hence a piece of the deep reality of the World-Wide Web is pulled into a time–space frame, the concepts’ meaning content is not identical with the images on the screen or the ink patterns on a printed page. Let us mention that we recently explored this aspect of the relation of concepts with their collapsed states, i.e. the images on the screen or the ink pattern on a printed page, to build a natural language processing type of theory for the World-Wide Web (Aerts et al. 2017). For more than one quantum entity the non spatiality becomes more evident since the Hilbert space modeling the joint entity is the tensor product of the Hilbert spaces modeling the individual entities. For example, for the case of two quantum entities this is $L^2({\mathbb {R}}^6)$ and the complex wave functions of 6 real variables that are not products of two complex wave functions of 3 real variables represent the entangled states, associated with the phenomenon which has been called quantum non-locality. In fact, it is easy to understand its origin, indeed, for two quantum entities it is on the level of the set of collapsed states that products are to be made, hence ${\mathbb {R}}^6={\mathbb {R}}^3 \times {\mathbb {R}}^3$, which just expresses that ‘two quantum entities can be found when a measurement to localise the joint entity is made’. This applies much less on the level of all states, also the non collapsed ones, as superpositions of product states not reducible to product states will exist for the joint entity. These are the complex wave functions of 6 variables not reducible to the product of two complex wave functions of 3 variables, which carry entanglement and give rise to the presence of non local correlations. The similarity of the situation of the World-Wide Web, its pages, and the stories, concepts and their combinations, it contains, shows again when we investigate how ‘joint concepts’ behave with respect to measurements which make their states collapse towards exemplars, i.e. more concrete—more localised in the meaning realm—concepts. We can easily identify entanglement experimentally by checking the violation of Bell’s inequality (Aerts 2010a). Let us analyse more specifically the realm of human knowledge like it is represented on the World-Wide Web with the aim of putting forward a possible understanding of the phenomenon of non-locality in the quantum physical realm.

Aerts (2013) studied in detail the way in which the words ‘and’, i.e. the ‘conjunction’ and ‘or’, i.e. the disjunction, appear in human language. It is fair to say that overall they both appear the same order of times in a corpus of texts such as the World-Wide Web. However the way in which they appear is quite different. The counts we made in Aerts (2013) took place by means of the Yahoo search engine on September 15, 2011, and let us give some of the results to illustrate the difference we want to point out. When two concepts with no obvious meaning connection are combined with the conjunction there are systematically more hits of this combination as compared with when the same concepts are combined with the disjunction. For example, combining the two concepts ‘horse’ and ‘house’, we found 12,500 hits for the combination ‘horse and house’ while only 4690 hits for the combination ‘horse or house’, which is a difference, expressed in terms of ratio, of $12{,}500/4690 \approx 2.6$. For the two concepts ‘flute’ and ‘bass’, we found 11,900 hits for the combination ‘flute and bass’ while 162 hits for the combination ‘flute or bass’, which corresponds to the ratio $11{,}900/162 \approx 73.4$. Hence we find systematically more hits for the conjunctive combination as compared to the disjunctive combination. Except if we consider concepts that have a special relation with each other. For example, for ‘laugh’ and ‘cry’ we find 297,000 hits for ‘laugh and cry’ while 779,000 hits for ‘laugh or cry’, hence giving the ratio $297{,}000/779{,}000 \approx 0.4$. For ‘death’ and ‘alive’ we find 149,000 hits for ‘death and alive’ while 13,100,000 hits for ‘death or alive’, hence giving rise to $149{,}000 / 13{,}100{,}000 \approx 0.01$. For ‘coffee’ and ‘tea’ we find 2,860,000 hits for ‘coffee and tea’ while 3,690,000 hits for ‘coffee or tea’, hence giving rise to a proportion $2{,}860{,}000 / 3{,}690{,}000 \approx 0.7$. We can easily understand these results, indeed, it is not difficult to imagine the many events where drinks are offered and ‘coffee or tea’ is an abundantly used combination. Even more so for the expression ‘death or alive’ and its much more abundant appearance as compared to the expression ‘death and alive’ and similarly for the difference in appearance between ‘laugh or cry’ and ‘laugh and cry’. These findings show that the conjunction combination are spread out quite equally amongst concepts that are not specifically connected by meaning while the disjunction combinations are concentrated amongst specific concepts. Disjunction is represented in the quantum formalism by superposition while conjunction is represented by product. What we just identified above in human language is hence a good representation of how entities are mathematically represented in the quantum formalism, superposition if different alternatives stand next to each other connected by a disjunction, i.e. one of them will be realised as a consequence of a collapse, and product, if different alternatives stand as realised jointly, or one after the other, and the tensor product of individual Hilbert spaces is indeed the structure describing joint entities.

We have explored this analogy between the structure of language and the structure of physical quantum entities in time–space in much more detail in Aerts (2013), but there is one aspect which we will put forward here. First of all we have to remark that more abstract concepts are filled with disjunctions of more concrete concepts, e.g. the concept *Animal* is the disjunction of *Dog*, *Cat*, ..., *Horse*, and the ‘...’ stands for all other animals. This means that in the quantum equivalent the state of *Animal* will be a superposition state of states of all the animals. Suppose now that we consider a specific story in which the concept *Animal* appears on several places in the text counting the story. If the state of *Animal* collapses to one of its superposition, e.g. to *Dog*, then in all places of the text counting the story *Animal* will collapse to *Dog* instantaneously within the meaning field of the story. The spread out of the text of the story in the realm of human language is the equivalent of the spread out in space of physical entities in the realm of quantum physics. Hence the simultaneous collapse of *Animal* to *Dog* all over the spread out text where it appears is the equivalent of the non local quantum action with respect to a correlation present in quantum entities localizable in distant regions of space. It indicates that a property which is quite easy to understand in language—namely that if a concept within the coherent whole of a story, changes its state, then this state change will happen in every place in the story where the concept appears—can make us understand why such a phenomenon also takes place for quantum entities in distant regions of space. Of course, this will only be the case within the coherent meaning whole of a story, and not all over the World-Wide Web. Similarly, for quantum entities spread over space, the type of instantaneous change in different possible widely separated places in space, provoked by a local change in one of the places, is only real, and is called non-locality, if the considered quantum entities are connected, i.e. part of a same coherent domain.

Relativity does not stop being influential at the atomic level, also subatomic entities and subnucleonic entities have their anti-particles and have masses that obey to the equation $E=mc^2$, something which is seen at each experiment in any particle collider. We have mentioned already that a quantum entity for most of its states is non spatial with respect to the three-dimensional Euclidian space. But, three-dimensional Euclidian space does function still for it as a canvas, like a piece of paper is a canvas for a text when ink is used to draw the concepts of the text in the form of words. On the subnucleonic level, hence for quarks and gluons, something more than non spatiality happens with respect to the three-dimensional Euclidian space. In the atomic and sub atomic realm, hence also for electrons, protons and neutrons, the symmetry that is predominant is represented by the special unitary group of two dimensions ${ SU}(2)$. For example, the spins of all particles to be found in this realm, electrons, protons, neutrons, atoms, molecules, including large ones, all have spins that are representations of ${ SU}(2)$. There is a deep similarity between ${ SU}(2)$ and ${ SO}(3)$, the group of all rotations in three-dimensional Euclidian space. They are almost isomorphic, and locally they are. Globally ${ SU}(2)$ is the universal covering group of ${ SO}(3)$. This is, by the way, the reason that spins of quantum entities show great similarity with rotations, which is also why they are called ‘spins’. If we consider the realm of quarks and gluons the predominant symmetry is no longer ${ SU}(2)$ but ${ SU}(3)$. There is no obvious group of symmetries in ${\mathbb {R}}^3$ which is similar to ${ SU}(3)$. What does this mean? Light can be shed on this situation by a recent generalisation we worked out for the Bloch representation which we called the extended Bloch representation (Aerts and Sassoli de Bianchi 2014). Let us first remark that the Bloch representation is the incarnation of the isometry between ${ SU}(2)$ and ${ SO}(3)$ and it is indeed built on a epresentation, already put forward by Henri Poincaré, of ${\mathbb {C}}^2$ by the points of the unit sphere of ${\mathbb {R}}^3$. A generalization of this representation can be worked out, of ${\mathbb {C}}^n$ by the points of a specific subset of the unit sphere of ${\mathbb {R}}^{n^2-1}$. For $n=2$, hence the case of electrons, protons, neutrons, atoms and molecules, where ${ SU}(2)$ is the predominant symmetry, we get $n^2-1=3$, which makes that ${\mathbb {R}}^3$ can be the canvas for the states of all the quantum entities of this realm. For $n=3$, hence the case of quarks and gluons, where ${ SU}(3)$ is the predominant symmetry, we get $n^2-1=8$. This means that we have to consider ${\mathbb {R}}^8$ if we want to find a canvas for these quantum entities. This necessity for ${\mathbb {R}}^8$ for quarks and gluons might well be the reason why quarks and gluons are not found in isolation witin our three dimensional Euclidean space.

We mentioned that no satisfactory theory of quantum gravitation exists at this moment. The reason why gravity resists the quantization procedures applied for the other forces and leading to the different quantum field theories unified in what is today called the Standard Model, is because gravity and the way it appears as a curvature of time–space due to mass in the general theory of relativity does not allow the quantization procedures used for the other forces to be applied. This is so because the other forces can be modeled as fields on a canvas of time–space while gravity involves changes in time–space itself. The quantization procedures that led to successful quantum field theories for the forces different from gravity consider time–space as the realm containing all of reality. If the time–space realm itself is affected by the force of gravity one might expect deep trouble of a paradoxical nature to follow. It is well possible that the new foundation for the theory of relativity we present here—where deep reality is supposed to be non temporal and non spatial and time–space is a means to model the different times of entities surfing through this deep reality, while the forces, including gravity, are means to ‘stay together’—opens the possibility to integrate also gravity with quantum theory. It is our plan to further reflect on this in the coming future, using our general operational-realistic conceptuality interpretation and explanation of quantum and relativity theories.

**Eplilogue**

I was still dazzled by the view that he had revealed to me and several thoughts crossed my mind, new questions and the desire to understand.

“Why is the relation between time and space hyperbolic?” I asked—it was, in my opinion, one of the most mysterious aspects of relativistic time–space, its hyperbolic relation. He smiled and I saw his eyes twinkle:

“Each entity evolves in its own pace of thoughtful dedication,” he answered, “but if another entity is forced to evolve in the realm of one specific entity, while this specific entity defines its own natural pace, it is less dedicated, that is understandable, is it not?” He frowned his brows pensively.

“Look,” he continued, “now you are reflecting at your pace about what I am trying to explain to you, if you want me to accompany you in your thoughts to your next question, I have to move more quickly than you, do you see?”

“Is what you call now ‘each entity’s own pace’ the speed of light?” I asked.

“Exactly,” he answered while he took a nip of his beer, “you are understanding, and I follow you in your reasoning.” He put his glass back on the table and moved his hand to accentuate his words:

“The natural pace is the speed of light in the realm of the material world, but it could in principle be another speed. What is important to note is that I have to keep up with you and stay with you and hence spend less time than you, if we measure the time in your realm.” I leaned forward on the table in reflection. The people in the center of the ballroom paused and a new piece of music was started.

“You see, these dancing couples also stay with us, although they are unaware of what we reflect about,” he continued, “so they have to move much more quickly still than I do, if we consider them in your realm of thought, and measure speed in your time.” His words made me look at the dancing crowd, as he went on:

“Of course, if we now watch the dancers, and hence stay with them in their realm, the opposite happens, we have to move faster, measured in their time, because the dancers move in their own dedicated pace.” I started to grasp and asked:

“And light itself is able to follow everything in a blink?” He smiled and paused and then spoke slowly:

“Not always, only when reflected by a mirror such that it can meet again, or, more subtly, when curved for a second meeting by gravity”.

****

He emptied his pipe in the ashtray on our table and put a new plug of tobacco. While he lighted a match and held it alongside the pipe head, he looked me in the eyes. His face wrinkled slightly as he pulled the fire into the tobacco and little clouds of smoke escaped his lips.

“It was not a good choice to call it ‘theory of relativity’,” he said smiling, “but taken into account the scientific spirit of that time, it was understandable.” I thought I knew at least partly what he meant, but I was eager to see whether I had really understood.

“Is it because of the principle of the constancy of the velocity of light in every reference frame that ‘theory of relativity’ was not a good name?” I asked.

“Exactly,” he answered, “but, there is much more to it than I and others had grasped at that time,” he continued. My curiosity was triggered, and I asked:

“What is this ‘much more’?”

“The velocity of light being a constant in every reference frame is a consequence of a very indirect nature and together with the focus on relativity it has merely obscured the understanding of what is really going on,” he said, while he looked at me and moved his eyebrows. While I leaned back in my chair intrigued by the expression on his face, he continued:

“Some of the things I will explain to you have been noted here and there, at least partly, in a fragmented way, by different analysis, but they have not been digested and definitely not been understood or taken seriously either. Let me mention one of these things, which you probably know.” He sat straight in his chair and made a puff of smoke holding his pipe.

“Whenever an object moves, the magnitude of its four-velocity is equal to the velocity of light, that is where the velocity of light enters the scene, and that is where we need to interpret its meaning.” I was amazed by the shift of focus he introduced when it comes to the velocity of light. In all analysis of the theory of relativity that I had read and reflected about, the velocity of light comes on the scene as ‘a maximum possible velocity and a constant independent of the reference frame where it is measured’, and of course—I had to pause and delve into my memory—I did know that if one calculates the magnitude of the four-velocity of an arbitrary object, one does find that it is equal to the velocity of light... but that is usually considered as a consequence and... indeed, I could not remember ever having read an explanation of this amazing equality.

“I see,” is the only reaction I managed to give while I was still reflecting. He looked at me and was visibly amused by my astonishment.

“What does it mean?” I asked, “that every object ‘moves’ with a four-velocity which has the magnitude of the velocity of light?”

“It means exactly what you have just said,” he whispered with a mysterious smile.

****

I remained silent. His pipe was loose in his mouth and little clouds of smoke filled the space between our faces.

“Do you mean that all material things ‘move’ at the velocity of light?” I asked, not aware that I was merely repeating my question.

“Exactly,” he answered.

“To move at the velocity of light,” I whispered trying to grasp its meaning, “is it ‘moving’ in the four-dimensional time–space continuum?” I asked.

“There is no four-dimensional time–space continuum,” he answered with a firm voice, and after a pause he continued:

“Things turn out to be quite different from what one was imagining in the years after relativity theory was born,” he said pensively.

“Can you explain,” I asked and I felt my attention sharpen to a level I had hardly ever experienced.

“Well,” he continued after taking a deep smoke, “I know that actually a lot of confusion exists, and that many have come to a view which they have called ‘the block universe’, where only the four-dimensional time–space continuum is believed to exist, and the passage of time is imagined to be an illusion.” He nodded his head:

“That is not a good way to go, don’t you think?” I felt that he did not expect an answer and hence did not change my listening attitude.

“We have to unravel the confusion that led to this view so we can come to see what is really going on,” he continued, “and we will see that a completely different situation unfolds, much more compatible with our intuition of what we believe reality to be, but at the same time containing new elements of deep strangeness.”

“You make me very curious,” I responded. He leaned backwards in his chair while my mind was surfing in fast speed through the content of my memory about the block universe view.

“Look,” he said and leaned further backwards so he could reach the menu-cards that were gathered on a little service table nearby. He grabbed one of the cards and opened it on the first page, where was written ‘Hors d’oeuvres’, and continued:

“We both look now over the list of appetizers on offer in this restaurant,” he said, while he moved his finger over the page, “but this does not mean that whatever the part of reality is that is generated by our looking is also contained in the booklet. The block universe view and the way to see reality as unchangeably described within the time–space continuum makes this mistake, to believe that if we read over the list of appetizers we are part of the list ourselves.” He paused and moved his finger again over the different printed lines:

“If we go over the list of appetizers we ‘move’ over the printed page, but of course, the printed page exists independently of our fingers. We move over it in one dimension, usually from top to bottom, but some might do it from bottom to top, or even jumping in a chaotic manner across the page, from one line to another. But it will always be in one dimension which can be related to by looking how time changes. It is however not a time dimension since it refers to a movement over the page of the menu-card. In a similar way, we, including all material entities of our universe, move over the deep reality at the velocity of light. We move in one dimension which is a line in the deep reality well described by the change of time.”

“Do you mean that we, and the rest of the material world are not part of the four-dimensional time–space, but move through it instead?” I asked. Now a mysterious smile had appeared on his face.

“Things are still quite different,” he said, “and perhaps you will be able to understand much better than you actually think.” He paused and then continued:

“Do you know why I was waiting in this ball room the whole afternoon to meet with you?” he asked, “I like to sit here and watch the dancing, but that was not the reason for me to spend so much time here, I came explicitly to meet with you.” I did not answer, but the change of subject and his words brought the notion of ‘non spatiality’ before my mind’s eye. This is a notion that we had introduced with my group in the 1980s of the forgoing century to explain aspects of quantum theory. He paused and watched me for a long time, while I too remained silent.

“Indeed,” he then said as if he had guessed my thoughts, “deep reality is not inside time–space. Time and space are constructions whose coming into existence is linked to the formation of matter.” I had to reflect a while on these words, and then asked:

“But you just said that the flow of time is real, and is related to ourselves and our surrounding material entities moving through deep reality at the velocity of light?”

“It is real, because brought into existence by our bodies, which are made of matter, and by all macroscopic material entities that accompany our bodies in this universe in a flight at the velocity of light. It is real but part of the specificity of our bodies and their accompanying material entities, hence what we are used to call ’the universe’, is just a vantage point with respect to deep reality,” and he paused and looked at me with a penetrating glance.
